# Development and Psychometric Validation of a Knowledge, Attitudes, and Practices Questionnaire on Pulmonary Embolism and Its Modifiable Risk Factors (PE-KAP-RO): Protocol for a Cross-Sectional Study in Adults from Western Romania

**DOI:** 10.3390/healthcare14213599

**Published:** 2026-10-23

**Authors:** Carina Gîb, Alina Anton, Raluca-Andreea Jupâneanț, Andreea-Adriana Neamțu, Laura Maghiar, Teodora Maria Bodog, Yasmina Laza, Teodor-Andrei Maghiar, Emanuel Ionuț Poplicean, Ion-Costel Epuraș, Valentin Cristian Iovin, Octavian Crețu, Daniel Florin Lighezan

**Affiliations:** 1 Doctoral School, “Victor Babeș” University of Medicine and Pharmacy Timișoara, Eftimie Murgu Square, No. 2, 300041 Timișoara, Romania; carina.gib@umft.ro (C.G.); emanuel.poplicean@umft.ro (E.I.P.); ion.epuras@umft.ro (I.-C.E.); valentin.iovin@umft.ro (V.C.I.); 2 Center of Advanced Research in Cardiology and Hemostasology, “Victor Babeș” University of Medicine and Pharmacy Timișoara, Eftimie Murgu Square, No. 2, 300041 Timișoara, Romania; dlighezan@umft.ro; 3 Department of Toxicology, “Victor Babeș” University of Medicine and Pharmacy Timișoara, Eftimie Murgu Square, No. 2, 300041 Timișoara, Romania; dolghi.alina@umft.ro; 4 Research Centre for Pharmaco-Toxicological Evaluation, “Victor Babeș” University of Medicine and Pharmacy Timișoara, Eftimie Murgu Square, No. 2, 300041 Timișoara, Romania; 5 Department of Pathology, Clinical County Emergency Hospital of Arad, Andrenyi Karoly Str., No. 2–4, 310037 Arad, Romania; 6 Department of Pathology, “Pius Brinzeu” Clinical County Emergency Hospital Timișoara, Liviu Rebreanu Boulevard, No. 156, 300723 Timișoara, Romania; 7 Department of Psycho-Neurosciences and Rehabilitation, Faculty of Medicine and Pharmacy, University of Oradea, Universității Str., No. 1, 410087 Oradea, Romania; laura.maghiar@uoradea.ro; 8 Department of Dermatovenerology, Clinical County Emergency Hospital Bihor, 410169 Oradea, Romania; 9 Department of Surgical Disciplines, Faculty of Medicine and Pharmacy, University of Oradea, Universității Str., No. 1, 410087 Oradea, Romania; bodog.teodoramaria@student.uoradea.ro (T.M.B.); teodor.maghiar@yahoo.com (T.-A.M.); 10 Bihor County Emergency Clinical Hospital, 410169 Oradea, Romania; 11 Doctoral School of Biomedical Sciences, University of Oradea, Universității Str., No. 1, 410087 Oradea, Romania; 12 Department of Cardiology, Affidea Timișoara, Coriolan Brediceanu Str., No. 10, 300011 Timișoara, Romania; dr.ylaza@gmail.com; 13 Department of Surgery, Pelican Hospital, Corneliu Coposu Str., No. 2, 410450 Oradea, Romania; 14 Center of Research and Innovation in Personalized Medicine of Respiratory Diseases (CRIPMRD), “Victor Babeș” University of Medicine and Pharmacy Timișoara, Eftimie Murgu Square, No. 2, 300041 Timișoara, Romania; 15 Oftalmo Sensory-Tumor Research Center–ORL (EYE-ENT), “Victor Babeș” University of Medicine and Pharmacy Timișoara, Eftimie Murgu Square, No. 2, 300041 Timișoara, Romania; 16 Department of Surgery I—Clinic of Surgical Semiotics & Thoracic Surgery, “Victor Babeș” University of Medicine and Pharmacy Timișoara, Eftimie Murgu Square, No. 2, 300041 Timișoara, Romania; octavian.cretu@umft.ro; 17 Center for Hepato-Biliary and Pancreatic Surgery, “Victor Babeș” University of Medicine and Pharmacy Timișoara, Eftimie Murgu Square, No. 2, 300041 Timișoara, Romania; 18 Department of Internal Medicine I, “Victor Babeș” University of Medicine and Pharmacy Timișoara, Eftimie Murgu Square, No. 2, 300041 Timișoara, Romania

**Keywords:** pulmonary embolism, venous thromboembolism, knowledge attitudes practices, questionnaire validation, psychometrics, item response theory, health literacy, modifiable risk factors, risk-factor burden, cross-sectional study

## Abstract

Background/Objectives: Venous thromboembolism (VTE), whose most severe manifestation is pulmonary embolism (PE), is the third most common acute cardiovascular disorder, and many of its risk factors≠—obesity, physical inactivity, smoking, and unfavorable dietary patterns—are modifiable. No validated instrument is currently available to assess knowledge, attitudes, and practices (KAP) regarding PE among adults in Romania, and the burden of self-reported thromboembolic risk factors in community settings has not been described with standardized measures. This protocol describes the development and psychometric validation of the PE-KAP-RO questionnaire, an integrated assessment tool comprising three scored KAP scales, three embedded validated lifestyle instruments, and an exploratory, unweighted VTE risk-factor burden index. No participants had been enrolled and no data had been collected at the time of submission; all procedures are therefore described as planned. Methods: The study comprises two phases. Phase 1 (development) includes qualitative concept elicitation with members of the target population, guideline- and theory-anchored item generation, expert content validation quantified by the content validity index, and pilot testing with cognitive debriefing (*n* ≈ 30). Phase 2 is a cross-sectional field validation in at least 450 adults (≥18 years) from Western Romania—with recruitment continuing toward 600 where feasible—through combined in-person (quota-guided) and online sampling that includes healthcare professionals, with test–retest re-administration in a subsample of at least 60 participants after 14 ± 4 days. Physical activity, diet, and nicotine dependence will be measured with the IPAQ-SF, MEDAS, and FTND. Sample size is justified separately for each analysis (precision of proportions, correlation and regression power, events per variable, split-sample factor analysis, item response theory calibration, and reliability estimation). Measurement properties will be evaluated against pre-specified acceptance criteria: item difficulty 0.20–0.80 (a screening range interpreted together with discrimination) and corrected item–total correlations ≥ 0.30; internal consistency (KR-20/Cronbach’s alpha and McDonald’s omega) 0.70–0.95; test–retest ICC ≥ 0.70 and item-level weighted kappa ≥ 0.60; structural validity by split-sample exploratory and confirmatory factor analysis (CFI/TLI ≥ 0.90, RMSEA ≤ 0.08, SRMR ≤ 0.08); item response theory calibration with formal model comparison and differential item functioning screening; construct validity requiring confirmation of ≥75% of a priori hypotheses; floor/ceiling effects < 15%; and measurement invariance across sex, age group, and administration mode. Expected Results: The study will deliver a validated Romanian-language instrument, a structured characterization of PE-related knowledge, attitudes, and preventive practices, and the distribution of self-reported risk factors in the study sample. Conclusions: PE-KAP-RO is intended as a standardized assessment tool for PE-related knowledge, attitudes, and practices; the risk-factor burden index is descriptive and does not estimate VTE probability; any future predictive tool would require a separate cohort followed for incident VTE. Reporting follows the STROBE recommendations and COSMIN-based methodology; the protocol is presented prior to participant enrollment.

## 1. Introduction

Venous thromboembolism (VTE), encompassing deep vein thrombosis (DVT) and pulmonary embolism (PE), is the third most frequent acute cardiovascular condition after myocardial infarction and stroke, with an annual incidence of approximately 1–2 cases per 1000 persons in Europe and North America and a substantial, still growing, global health burden [1,2,3,4]. PE is the potentially fatal manifestation of VTE and remains an important and frequently under-recognized cause of cardiovascular death; roughly one in five patients with VTE dies within the first year, often from associated conditions, and a considerable proportion of survivors develop persistent functional limitations described as post-PE syndrome [2,3,5,6]. A recent analysis of the World Health Organization mortality database (2001–2023, 73 countries) documented a global decline in age-standardized PE mortality but persistent or rising rates in low- and middle-income settings, underscoring the continued importance of prevention [7].

According to Virchow’s triad—venous stasis, endothelial injury, and hypercoagulability—VTE arises from the interplay of non-modifiable (advancing age, sex, inherited thrombophilia, prior VTE, malignancy) and modifiable, behavior-related risk factors [1,2,3]. Obesity, which affects nearly 60% of adults in the WHO European Region, is a prothrombotic, low-grade inflammatory state associated with altered adipokine and cytokine profiles [8,9,10]; physical inactivity is associated with an increased VTE risk in meta-analyses of prospective cohorts [11]; smoking, whose effect on VTE risk is strongest in combination with cancer, remains highly prevalent in Central and Eastern Europe [12,13]; and anti-inflammatory dietary patterns and ultra-processed food intake have been prospectively associated with lower and higher VTE risk, respectively [14,15], while Mediterranean-type diets are an established component of cardiovascular prevention [16]. Transient or situational factors—prolonged immobilization and hospitalization, major surgery and trauma, long-distance travel, pregnancy and the postpartum period, hormonal therapy, and active cancer—complete the picture [17,18,19,20]. Because modifiable factors are targets for primary prevention, their distribution and the public’s awareness of them are of direct public-health relevance [3,4].

Knowledge–attitudes–practices (KAP) surveys are a well-established approach for measuring health literacy and behavior [21] and, when applied to thromboembolic disease in hospitalized and community populations, have consistently found limited knowledge of DVT/PE warning signs and prevention [22,23,24]; similar gaps have been described for cardiovascular risk factors in general [25]. The acceptability of preventive dietary change, such as reducing red meat intake, has recently been examined in Romanian adults, providing local context for the dietary items [26]. The Health Belief Model, which links perceived severity, susceptibility, benefits, self-efficacy, and cues to action to preventive behavior, provides a theoretical framework for their attitudinal dimension and continues to underpin instrument development [27,28].

However, KAP studies are frequently limited by ad hoc questionnaires whose measurement properties are never formally evaluated, which undermines the comparability and interpretability of their results. Instruments with documented measurement properties in this field address other populations or constructs—VTE-prevention knowledge and practice among nurses [29] or anticoagulation knowledge among treated patients [30]—whereas public-awareness surveys, including the multinational survey conducted for World Thrombosis Day [31], used descriptive questionnaires without formal measurement evaluation. Consensus-based standards for the selection and evaluation of health measurement instruments (COSMIN) and best-practice guidance for scale development define how new instruments should be developed, evaluated, and reported [32,33,34]. To our knowledge, no psychometrically validated instrument is available to assess PE-related knowledge, attitudes, or practices among adults in Romania, the burden of self-reported thromboembolic risk factors in the community has not been described with standardized measures, and existing VTE risk assessment models were developed for hospitalized patients and are not designed for self-administration [35].

The primary objective of this study is therefore to develop and psychometrically validate the PE-KAP-RO questionnaire—an integrated instrument assessing knowledge, attitudes, and preventive practices regarding PE and its modifiable risk factors—in adults from Western Romania. Secondary objectives are (i) to characterize the level and sociodemographic correlates of PE-related knowledge, attitudes, and practices; (ii) to describe, using validated instruments, the distribution of modifiable risk factors (obesity, physical inactivity, dietary pattern, and smoking) in the study sample; (iii) to describe individual self-reported VTE risk factors and an exploratory, unweighted risk-factor burden index, together with their associations with knowledge, attitudes, and practices, as descriptive groundwork for future, separately designed predictive research; and (iv) to identify subgroups with lower awareness and less favorable preventive behavior that could benefit from targeted intervention.

## 2. Materials and Methods

### 2.1. Study Design and Setting

**Figure 1:**
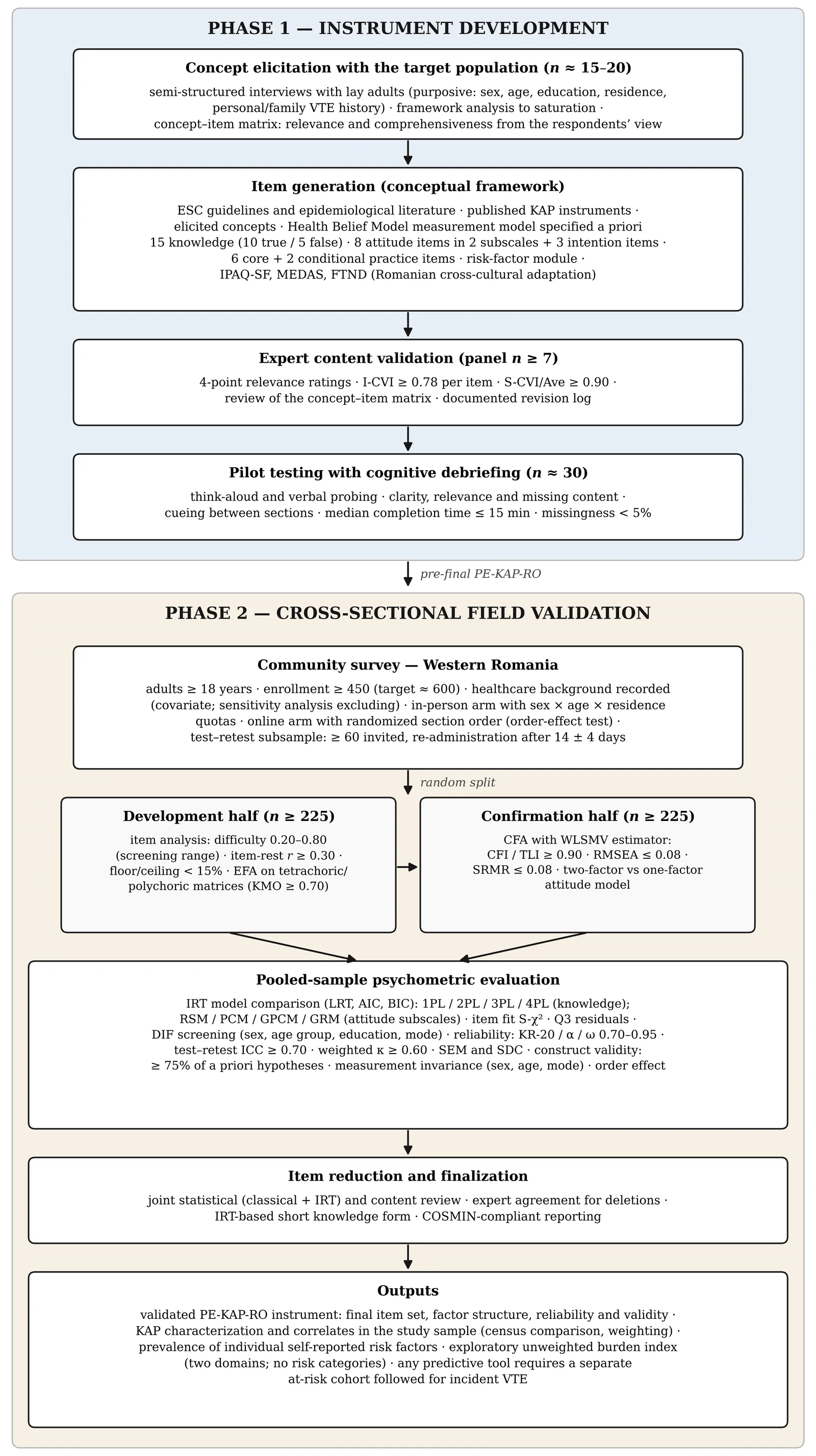
Two-phase design of the PE-KAP-RO development and validation study. CVI—content validity index; EFA/CFA—exploratory/confirmatory factor analysis; KMO—Kaiser–Meyer–Olkin; WLSMV—weighted least squares with mean and variance adjustment; IRT—item response theory; 1PL/2PL/3PL/4PL—one-/two-/three-/four-parameter logistic models; RSM—rating scale model; PCM—partial credit model; GPCM—generalized partial credit model; GRM—graded response model; LRT—likelihood-ratio test; AIC/BIC—Akaike/Bayesian information criteria; VTE—venous thromboembolism; DIF—differential item functioning; KR-20—Kuder–Richardson formula 20; ICC—intraclass correlation coefficient; SEM—standard error of measurement; SDC—smallest detectable change; KAP—knowledge, attitudes, and practices.

This article describes the protocol of a planned study. At the time of submission, no participants had been enrolled and no data had been collected; all procedures are therefore described as planned. The study is a cross-sectional, observational instrument-development and validation study that will be conducted in Western Romania (Timiș County and the surrounding region) in two phases. Phase 1 (development) will comprise qualitative concept elicitation with members of the target population, item generation, expert content validation, and pilot testing with cognitive debriefing. Phase 2 (field validation) will be a community-based cross-sectional survey. Participants will be recruited in person in community and outpatient settings, guided by demographic quotas, and online through institutional and social-media channels, in order to reach a broad cross-section of adults in the region. The study is coordinated by the Doctoral School of Medicine of the “Victor Babeș” University of Medicine and Pharmacy Timișoara. Reporting will follow the STROBE statement for cross-sectional studies [36], and the design, evaluation, and reporting of measurement properties will follow COSMIN-based methodology [32,34]. The overall design is summarized in Figure 1.

### 2.2. Study Population and Eligibility Criteria

Eligible participants are community-dwelling adults meeting the following criteria.

Inclusion criteria: age ≥ 18 years; residence in the study region; ability to understand Romanian; and provision of informed consent.

Exclusion criteria: inability or unwillingness to provide consent, or incomplete responses to core KAP items.

Healthcare professionals and health-sciences students are part of the adult population and are therefore eligible. Their professional background will be recorded (item A8) and taken into account in three ways: as a covariate in all multivariable models, by reporting descriptive results separately for participants with and without a healthcare background, and through a sensitivity analysis that excludes participants with a healthcare background (Section 2.7). The target population is therefore defined as adults living in Western Romania. Given the recruitment strategy (Section 2.6), the findings will be generalizable mainly to adults in the region who can be reached through community, outpatient, and online channels; the composition of the sample will be compared with census data.

Four further groups will take part in specific phases of the study: (i) approximately 15–20 adults from the target population, who will take part in qualitative concept-elicitation interviews before the item pool is finalized (Section 2.4.1); (ii) an expert panel of at least seven members (internal medicine, cardiology, pulmonology, epidemiology/public health, primary care, and biostatistics/psychometric methodology), who will assess content validity; (iii) a pilot sample of approximately 30 adults from the target population, who will complete the pre-final version with cognitive debriefing (pilot data will not be included in the final analysis); and (iv) a consenting subsample of field-phase participants, who will complete the questionnaire a second time after 14 ± 4 days to assess test–retest reliability. This interval is long enough to limit recall and short enough for the measured constructs to be assumed stable [37]. At least 60 participants will be invited, so that at least 50 complete pairs of administrations are available for analysis (Section 2.3) [38].

### 2.3. Sample Size and Power Considerations

The sample size was justified separately for every planned analysis; the resulting requirements, assumptions, and provisions are summarized in Table 1. The binding requirement is the descriptive-epidemiological aim, inflated for invalid questionnaires, giving a minimum enrollment of 450; recruitment will continue toward approximately 600 participants where feasible, which will improve precision and strengthen the item response theory, differential item functioning, and invariance analyses. The adequacy of the planned sample for the latent-variable analyses, and its limits, are discussed at the end of this section and in Section Limitations, Anticipated Risks, and Their Management.

**Estimation of proportions.** For the primary descriptive aim of estimating the proportion of participants with adequate knowledge of PE, the standard formula for a single proportion, *n = Z^2^·p·(1 − p)/d^2^*, with *Z* = 1.96 (95% confidence), the most conservative assumption *p* = 0.50, and an absolute precision *d* = 0.05, yields *n* = 384; allowing for approximately 15% incomplete or invalid questionnaires gives the enrollment target of at least 450. With 450 analyzable questionnaires, the achieved precision is ±4.6 percentage points, improving to ± 4.0 with 600. These figures express statistical precision only; they do not account for selection bias arising from non-probability sampling, which is addressed through quotas, census comparison, and weighting (Section 2.6 and Section 2.7) and limits generalization to the population described in Section 2.2.

**Correlation and regression analyses.** A total of ≈347 participants provides 80% power (two-sided *α* = 0.05) to detect correlations as small as |*r*| = 0.15 between KAP scores and continuous lifestyle indicators. For multivariable linear regression with up to 15 candidate predictors, ≈ 395 participants provide 80% power to detect a small incremental contribution of an individual predictor (Cohen’s *f^2^* = 0.02). For logistic regression modeling adequate knowledge, an anticipated prevalence of 30–50% yields ≥ 135 events at *n* = 450, satisfying the events-per-variable criterion (≥10) for models with up to 12 predictors; the more exacting criteria proposed for prediction-model development [39] would apply to any future, separately designed predictive study (Section 2.5).

**Factor analysis and IRT.** The reflective part of the instrument comprises 23 items: 15 knowledge items and 8 attitude-subscale items. With at least 450 valid questionnaires, the sample can be split at random into a development half and a confirmation half of at least 225 participants each. Within each half, this gives approximately 15 respondents per item for the knowledge scale and more than 25 per item for the attitude items, which will be factor-analyzed separately. These ratios exceed the COSMIN benchmark of at least seven times the number of items (and at least 100 participants) and the sample sizes most often used in published validation studies [32,33,40]. Item response theory (IRT) calibration (Section 2.8) will use the full pooled sample. Several hundred respondents are recommended for IRT, and at least 500 are desirable for stable two-parameter estimates; three- and four-parameter models require larger samples and will therefore be estimated with priors and interpreted cautiously [41,42]. This is a further reason for extending recruitment toward 600 participants.

**Adequacy of the sample for the latent-variable analyses.** Respondent-to-item ratios are only a rough guide. The sample needed for factor analysis and IRT depends mainly on the strength of the factor loadings, the number of items per factor, and the response format; dichotomous or highly skewed items require larger samples than continuous indicators (Section Limitations, Anticipated Risks, and Their Management). The planned split halves (at least 225 participants each) are expected to be adequate if standardized loadings are moderate to high (approximately ≥0.50) and each factor has at least four indicators, as planned for the knowledge scale (15 items) and for each attitude subscale (4 items). They may be insufficient if loadings are weak, if many knowledge items are very easy, or if fewer than 450 valid questionnaires are obtained. Before field testing, a Monte Carlo simulation of the hypothesized factor models will therefore be run in R, assuming loadings of 0.40–0.70 and the expected item distributions, to estimate the probability of convergence, parameter bias, and power with 225 and 300 participants per half. For IRT, a sample of 450–600 participants is adequate for the one- and two-parameter logistic models and for the graded response model, whereas three- and four-parameter models will be treated as exploratory [41,42]. The differential item functioning (DIF) and measurement-invariance analyses compare subgroups of roughly 150–300 participants. They will therefore be interpreted as screening analyses, with effect sizes reported alongside significance tests, and comparisons involving fewer than 150 participants per group will be reported as exploratory only. Pre-specified fallback strategies for each of these situations are described in Section Limitations, Anticipated Risks, and Their Management.

**Reliability.** Following simulation-based recommendations for reliability studies, approximately 50 paired administrations allow the ICC of each scale to be estimated with a 95% confidence interval half-width of about 0.10 when the true ICC is ≥0.80; at least 60 participants will therefore be invited into the retest subsample to offset attrition [38]. The pilot sample of ≈ 30 follows accepted practice for feasibility and cognitive testing [33].

### 2.4. The PE-KAP-RO Instrument: Development, Structure, and Scoring

#### 2.4.1. Concept Elicitation, Conceptual Framework, and Item Generation

Before the item pool is finalized, qualitative concept-elicitation interviews will be conducted with approximately 15–20 adults from the target population, purposively sampled for variation in sex, age group, educational level, and urban/rural residence and including both people with a personal or family history of VTE and people without any contact with the condition. Semi-structured individual interviews (or small focus groups) will explore what participants know and believe about blood clots and PE, which risk factors and warning signs they consider relevant, what they do or would do to prevent them, where they obtain health information, and which barriers to prevention they perceive. Interviews will be audio-recorded, transcribed, and analyzed with a framework approach; recruitment will continue until no new relevant concept emerges in two consecutive interviews. Elicited concepts will be mapped against the draft item pool in a concept–item matrix: concepts not covered will generate new or reworded items, and draft items whose content participants do not recognize as relevant will be flagged for the expert panel, in line with good-practice recommendations for establishing the content validity of new patient- and public-reported instruments [43,44].

In addition to the concept-elicitation findings, items will be generated from three sources: (i) current European guidelines and epidemiological literature on VTE/PE and its risk factors [1,2,3]; (ii) previously published KAP instruments addressing thromboembolic disease [22,23,24]; and (iii) the Health Belief Model [27,28], which structures the attitude section according to the measurement model specified in Section 2.4.4. The draft knowledge scale was deliberately constructed with a balance of true and false statements (10 true, 5 false) to limit acquiescence bias, and all items were drafted at a readability level appropriate for the general population. The instrument is administered in Romanian; the full English version is provided in Appendix A and the scoring algorithm in Appendix B; the Romanian participant form as administered, including the informed-consent section, is provided as Supplementary File S1, and its English translation as Supplementary File S2.

#### 2.4.2. Content Validity

Each expert will rate the relevance of every item to its intended construct on a 4-point scale (1 = not relevant to 4 = highly relevant) and will comment on clarity, wording, and comprehensiveness (missing content). Together, these cover the three dimensions of content validity distinguished by COSMIN: relevance, comprehensiveness, and comprehensibility [45]. The item-level content validity index (I-CVI) and the scale-level average (S-CVI/Ave) will be calculated. Items with an I-CVI ≥ 0.78 will be retained, and items below this threshold will be revised or deleted; the target S-CVI/Ave is ≥0.90 [46]. A revision log will link every change to the panel’s feedback. The experts will also review the concept–item matrix, so that comprehensiveness is judged against both the literature and the concepts raised by lay participants.

#### 2.4.3. Face Validity and Pilot Testing

The pre-final version will be piloted with approximately 30 members of the target population, selected to vary in age and educational level. Cognitive debriefing (think-aloud and verbal probing) will assess how each item is understood, how answers are retrieved and judged, and how they are mapped onto the response options. Participants will also rate the clarity of each item and the personal relevance of each section, and will be asked whether important aspects of preventing or recognizing blood clots are missing; in this way, relevance and comprehensiveness will also be assessed from the respondents’ perspective. Additional probes will check whether earlier sections suggest answers to later ones. The pre-specified feasibility criteria are: at least 80% of participants rating each item as clear, a median completion time of no more than 15 min, and less than 5% missing responses per item. Items that do not meet these criteria will be reworded and, if substantially changed, re-evaluated by the expert panel. If the median completion time exceeds 15 min, lower-priority items identified during debriefing will be removed before field testing.

#### 2.4.4. Structure and Scoring

The final instrument (Appendix A) will comprise nine modules, whose structure and scoring are summarized in Table 2. The modules will be administered in a fixed order: sociodemographic data and risk factors (A–B), attitudes (C), preventive practices (D), knowledge (E), and the lifestyle instruments (F–I). Placing the knowledge statements, which name symptoms and risk factors, after the attitude and practice sections prevents them from acting as educational cues for these responses [47]; possible order effects will also be tested experimentally in the online arm (Section 2.6).

**Table 2:** Structure and scoring of the PE-KAP-RO instrument.

Module	Items	Format	Score Range	Provisional Interpretation
A. Sociodemographic and anthropometric	8	Mixed (categorical, numeric)	—	Descriptive; BMI derived
B. Personal and family risk factors	10	Yes/no/don’t know; checklists	—	Descriptive; feeds burden index (Table 3)
C. Awareness screening and attitudes (Health Belief Model)	1 + 11	Screening item (yes/no); 5-point Likert (1–5)	Two subscales, 4–20 each; 3 single items	Per subscale ≥16 favorable; 12–15 neutral; <12 unfavorable; intention items reported individually
D. Preventive practices	6 core + 2 conditional	Ordinal 0–2; conditional items with “not applicable” option	0–12 (core items)	≥75% good; 50–74% moderate; <50% limited; conditional items descriptive
E. Knowledge	15	True/false/don’t know (10 true, 5 false)	0–15	<8 low; 8–11 moderate; ≥12 high; adequate ≥11 (≥70%)
F. Physical activity (IPAQ-SF)	7	Days and minutes, past 7 days	MET-min/week	Low/moderate/high (official IPAQ scoring rules)
G. Diet (MEDAS)	14	Dichotomous (1 point per criterion)	0–14	≤5 low; 6–9 moderate; ≥10 high adherence
H. Smoking (status, pack-years; FTND for current smokers)	1 + 6	Categorical; FTND ordinal	FTND 0–10	0–2 very low … 8–10 very high dependence
I. Alcohol intake	3	Categorical; units/week	—	Descriptive
Exploratory risk-factor burden index (derived)	14 elements in 2 domains	Unweighted counts of self-reported factors	Clinical/situational 0–10; lifestyle 0–4	Domains reported separately, not summed; no risk categories

**Table 3:** Exploratory VTE risk-factor burden index: elements, domains, and evidence basis (unweighted).

Self-Reported Element (Source Item)	Domain	Evidence Basis
Personal history of DVT/PE (B1)	Clinical	Established factor; Padua, Caprini
Known thrombophilia (B3)	Clinical	Padua, Caprini
Active cancer, or treated within the last 5 years (B4)	Clinical	Padua, Caprini (broader self-report definition)
Major surgery or trauma/fracture, past 3 months (B5)	Clinical	Padua, Caprini
Immobilization/hospitalization ≥ 3 days, past 3 months (B6)	Clinical	Padua (reduced mobility), Caprini
Age ≥ 60 years (A1)	Clinical	Age gradient of incidence; model thresholds differ
First-degree family history of VTE (B2)	Clinical	Caprini
Hormonal contraception/replacement, or pregnancy/postpartum (B8)	Clinical	Padua, Caprini [19]
Long-distance travel > 4 h, past month (B7)	Clinical (situational)	Epidemiological evidence [18]
Varicose veins/chronic venous insufficiency (B9)	Clinical	Caprini
Obesity, BMI ≥ 30 kg/m^2^ (A6)	Lifestyle	Padua, Caprini [8]
Current smoking (H1)	Lifestyle	Epidemiological evidence [12]
Low physical activity (IPAQ-SF category low)	Lifestyle	Epidemiological evidence [11]
Low Mediterranean diet adherence (MEDAS ≤ 5)	Lifestyle	Epidemiological evidence [14,15]

The index is an exploratory count of self-reported risk factors. Elements are unweighted; the clinical/situational (0–10) and lifestyle (0–4) domain counts are reported separately and not summed, and no risk categories are defined. Equal counts do not imply equal clinical importance, and the index does not estimate the probability of VTE (Section 2.5). DVT—deep vein thrombosis; PE—pulmonary embolism; VTE—venous thromboembolism; BMI—body-mass index; IPAQ-SF—International Physical Activity Questionnaire–Short Form; MEDAS—Mediterranean Diet Adherence Screener.

The three purpose-built KAP sections will be scored as follows. **Knowledge** (15 true/false/”don’t know” statements): one point is given for each correct answer, and “don’t know” is scored as incorrect (total 0–15). Provisional bands are <8 low, 8–11 moderate, and ≥12 high knowledge, and “adequate knowledge” is pre-specified as ≥11 points (≥70% correct). **Attitudes**: the measurement model is specified before data collection. Eight 5-point Likert items (1–5) form two correlated reflective subscales based on the Health Belief Model: perceived threat (AT1–AT4; two severity and two susceptibility items) and perceived benefits and self-efficacy of prevention (AT5–AT8; two benefit and two self-efficacy items). Each subscale is scored 4–20, and a score ≥ 16 (item mean ≥ 4) is considered favorable; no total attitude score is computed. Three further items—willingness to change lifestyle (AT9), information-seeking (AT10), and care-seeking intention (AT11)—measure behavioral intentions rather than a common latent attitude. They will be reported individually and used in construct-validity testing but not summed. **Practices**: preventive practice is defined as self-reported habitual behaviors that apply to most adults and that counteract venous stasis, support weight control and regular medical review, or reflect active engagement with information on VTE prevention. The practice index comprises six core items that apply to all respondents (PR1–PR6: breaking up prolonged sitting, moving the legs and feet during prolonged sitting, monitoring body weight, attending regular check-ups, seeking information, and discussing one’s own risk of blood clots with a health professional). Each item is scored 0–2, and the items are summed (0–12; prorated only when a single item is missing). The total is also expressed as a percentage: ≥75% good, 50–74% moderate, and <50% limited preventive practice. Two situation-specific items (PR7, movement during long journeys; PR8, adherence to recommended thromboprophylaxis) apply only to participants who have been in these situations; they are not part of the index and will be reported descriptively among those to whom they apply. The index is formative: its items are not assumed to reflect a single latent variable [48]. All bands are provisional and will be re-examined against the empirical distributions in the validation sample before they are finalized.

Physical activity, diet, and nicotine dependence will be measured with instruments whose measurement properties have been confirmed in European and international populations: the International Physical Activity Questionnaire–Short Form (IPAQ-SF; MET-minutes/week; low/moderate/high) [49,50,51], the 14-item Mediterranean Diet Adherence Screener (MEDAS; 0–14; low/moderate/high adherence) [52,53,54], and, for current smokers, the Fagerström Test for Nicotine Dependence (FTND; 0–10) [55,56]. Pack-years and alcohol intake will be recorded for all participants; alcohol is recorded because its association with VTE is uncertain [57]. Sociodemographic and anthropometric data (self-reported height and weight for body-mass index, BMI, and waist circumference where known), professional background (item A8), and personal and family risk factors complete the instrument.

The embedded instruments will be scored according to their original instructions, applied identically in the text, tables, and appendices. For the IPAQ-SF, bouts < 10 min are excluded, time per activity type is truncated at 180 min per day, and cases reporting >960 min per day in total are excluded; activity is classified as high (vigorous activity on ≥3 days accumulating ≥1500 MET-min/week, or ≥7 days of any combination of walking, moderate, and vigorous activity accumulating ≥3000 MET-min/week), moderate (not high, and ≥3 days of vigorous activity of ≥20 min/day, or ≥ 5 days of moderate activity and/or walking of ≥30 min/day, or ≥5 days of any combination accumulating ≥600 MET-min/week), or low (neither criterion met) [51]. MEDAS is scored 0–14 and categorized as low (≤5), moderate (6–9), or high (≥10) adherence [54,58]. The FTND is scored 0–10 and categorized as very low (0–2), low (3–4), moderate (5), high (6–7), or very high (8–10) dependence [55]. To our knowledge, no Romanian version of the IPAQ-SF, MEDAS, or FTND with published measurement properties is available; a recent Romanian study of Mediterranean diet adherence likewise relied on an ad hoc translation of MEDAS [58]. The draft Romanian versions (Supplementary File S1) were prepared by forward translation. During Phase 1, they will undergo a second independent forward translation, reconciliation, blinded back-translation, expert-committee review, and cognitive debriefing within the pilot, following established cross-cultural adaptation guidelines [59,60]; all adaptations (for example, locally familiar food examples, with scoring criteria unchanged) will be documented. Test–retest reliability of the three Romanian versions will be estimated in the retest subsample, and the internal consistency and unidimensionality of the FTND will be assessed among current smokers, so that these versions are reported with their own measurement evidence. The three instruments are freely available for non-commercial research use, and no licensing permission is required.

The primary outcome of the field phase is the psychometric performance of the PE-KAP-RO scales against the criteria pre-specified in Section 2.8. Secondary outcomes are the knowledge, attitude, and practice scores, the prevalence of each self-reported risk factor, and the distribution of the exploratory risk-factor burden index described below.

### 2.5. Individual Risk Factors and Exploratory VTE Risk-Factor Burden Index

The individual risk factors recorded in Modules A, B, F, G, and H will be analyzed primarily one by one, as sample prevalences with 95% confidence intervals. Because the elements, definitions, and thresholds used here differ from those of established risk assessment models—for example, the age threshold, the broader self-report definition of cancer, and the addition of travel and lifestyle variables—any composite derived from them constitutes a new construct rather than an adaptation of the Padua Prediction Score or the Caprini model, which were developed for hospitalized patients and continue to be evaluated in that setting [35,61,62,63,64]. For this reason, no weighted score is derived; instead, an exploratory risk-factor burden index (Table 3) is defined as unweighted counts of self-reported factors in two domains, which will be reported separately and not summed: a clinical/situational domain (10 elements; 0–10) and a lifestyle domain (4 elements; 0–4). Element selection draws on these models and on the epidemiological literature [3], but no weights are assigned, so that several modest lifestyle factors cannot numerically equal a previous VTE or active cancer.

The intended use of the index is deliberately limited. First, it is a descriptive, exploratory measure of self-reported risk-factor burden: it does not estimate the probability of VTE, no low/intermediate/high risk categories are defined, and no individual risk feedback is provided. Second, in the present study, its evaluation is restricted to feasibility (completeness and “don’t know” rates), distribution, and associations with knowledge, attitudes, practices, and lifestyle indicators; the cross-sectional design cannot establish predictive validity. Third, any future predictive tool would require a separately designed study—a cohort of adults free of VTE at baseline and followed for incident, objectively confirmed VTE (for example, through linkage to hospital records), in which weights are estimated and discrimination and calibration are evaluated according to contemporary prediction-model standards [39,65], as has been done for community VTE risk algorithms derived from large prospective cohorts [66]. Prognostic scores for patients with established PE, such as the PESI and sPESI [67,68], predict outcomes after PE has occurred and are therefore not an appropriate reference for the criterion validation of a susceptibility measure.

### 2.6. Data Collection Procedures

After giving informed consent, participants will complete the questionnaire once, either on paper (self-administered, with a trained assistant available to answer questions) or through a secure online form. Completion is expected to take approximately 15 min; this will be verified in the pilot. Responses will be pseudonymized with a numeric participant code at entry. Participants in the test–retest subsample will be contacted again through their preferred channel and will complete the identical instrument, in the same mode, after 14 ± 4 days; no feedback on their answers will be given between the two administrations. Score equivalence between paper and electronic administration has been repeatedly demonstrated for well-constructed instruments [69]; nevertheless, the administration mode (paper vs. online) will be recorded for all participants and formally tested as a possible source of measurement non-invariance (Section 2.8). Automated and manual data-quality checks will flag implausibly short completion times (<5 min), straight-lining on the attitude items, and inconsistent anthropometric entries. Flagged records will be reviewed and, if judged invalid, excluded, with the reasons documented.

**Recruitment quotas.** In-person recruitment will take place in community settings (e.g., workplaces, community centers, pharmacies, and public events) and in outpatient waiting areas. It will be guided by predefined quotas for sex, age group (18–39, 40–59, and ≥60 years), and area of residence (urban/rural), set in proportion to the adult population of the region in the most recent census [70]. Progress toward the quotas will be monitored weekly, and recruitment will be redirected toward under-represented groups. The composition of the online sample cannot be controlled in advance, but it will be monitored, and targeted distribution through local community and seniors’ networks will be used when groups are under-represented. Because the final sample will still be a non-probability sample [71], all estimates will be presented as estimates for the study sample. The census comparison and the weighted sensitivity analyses (Section 2.7) will indicate the likely direction and size of selection effects.

**Question-order effects.** In the online arm, the survey platform will randomly assign participants (1:1) to the standard section order (attitudes and practices before knowledge) or to a knowledge-first order. Comparing attitude and practice scores between the two groups will show whether the knowledge statements act as an educational cue (Section 2.7). If a meaningful difference (|g| ≥ 0.20) is found, the psychometric analyses of the attitude and practice sections will be repeated in respondents who received the standard order only.

### 2.7. Statistical Analysis: General Plan

Analyses will be performed in R (R Foundation for Statistical Computing, Vienna, Austria), with two-sided *α* = 0.05, using the following packages for each procedure: psych for descriptive item statistics, KR-20, Cronbach’s alpha, McDonald’s omega, tetrachoric/polychoric correlations, parallel analysis, and exploratory factor analysis; lavaan for confirmatory factor analysis with the WLSMV estimator and the exploratory structural equation model [72]; semTools for multi-group measurement invariance testing; mirt for IRT calibration, model comparison, item fit, local dependence (Q3), and IRT likelihood-ratio DIF tests [73]; lordif for ordinal logistic regression DIF [74]; irr for weighted kappa and intraclass correlation coefficients; mice for multiple imputation [75]; survey for raking and weighted estimation; rms for restricted cubic splines; and boot for bootstrap confidence intervals. IBM SPSS Statistics (version 29) may be used for descriptive cross-checks only. Continuous variables will be summarized as the mean ± standard deviation or median (interquartile range) according to the distribution (Shapiro–Wilk test), and categorical variables as counts and percentages. Group comparisons will use the t-test or Mann–Whitney U test for two groups and ANOVA or Kruskal–Wallis for more than two, with *χ^2^* or Fisher’s exact test for categorical variables. Every comparison will be reported with its effect size and 95% confidence interval (Hedges’ g or the rank-biserial correlation for two-group contrasts; *η^2^* or *ε^2^* for multi-group contrasts; Cramér’s V for contingency tables) rather than with *p*-values alone. Associations between KAP scores and continuous lifestyle indicators (BMI, IPAQ MET-minutes, MEDAS) will be examined with Pearson or Spearman correlation. Because the descriptive-epidemiological analyses involve a family of exploratory comparisons, the Benjamini–Hochberg procedure will control the false discovery rate at 5% within each pre-defined family, with unadjusted *p*-values also reported for transparency.

Multivariable linear regression will be used to identify independent correlates of the knowledge and practice scores, and logistic regression will model adequate knowledge (≥11/15) as a function of sociodemographic and lifestyle variables. Model diagnostics are pre-specified: multicollinearity (variance inflation factor < 5), residual analysis, and assessment of non-linearity for age and BMI with restricted cubic splines. As a robustness check, regression coefficients will be accompanied by bootstrap 95% confidence intervals (2000 resamples). An exploratory structural equation model will test the expected pathway from knowledge to attitudes to practices, with bias-corrected bootstrap confidence intervals for indirect effects. Missing data will be described; the primary models will use complete-case analysis, and multiple imputation by chained equations (*m* = 20, predictive mean matching) will be used as a sensitivity analysis [76].

Several additional analyses address the sampling strategy and the design. First, the sociodemographic composition of the sample will be compared with regional census data [70], and a post-stratification (raking) weighting analysis on age group, sex, residence, and educational level will be performed as a sensitivity analysis; weighting corrects only for these characteristics and not for unmeasured selection (for example, interest in health or digital literacy). Second, professional background (A8) will be entered as a covariate in all multivariable models; descriptive estimates will be reported overall and by professional background, and a sensitivity analysis will exclude participants with a healthcare background. Third, question-order effects will be tested in the randomized online arm by comparing attitude-subscale and practice scores between the two orders (Hedges’ g with 95% confidence interval), and order will be entered as a covariate in sensitivity analyses. Finally, individual self-reported risk factors will be summarized as sample prevalences with 95% confidence intervals, overall and by sex and age group, and the two domain counts of the burden index will be summarized as distributions (median, interquartile range, and frequency of each count), without categorization.

### 2.8. Psychometric Evaluation Plan

The measurement properties of the PE-KAP-RO scales will be evaluated in the sequence and against the pre-specified acceptance criteria summarized in Table 4; the attitude subscales will be analyzed as reflective scales, whereas the practice index is formative and the intention items are single-item indicators.

**Item analysis.** For each knowledge item, the difficulty index (proportion of correct answers) will be compared with a screening range of 0.20–0.80. The knowledge scale has two purposes: to describe awareness, for which easy items covering essential content (such as warning signs) are informative, and to distinguish between respondents with different levels of knowledge. The range will therefore be used to flag items for review rather than as a rule for deleting them. Items outside the range (up to 0.90 for content-critical items) will be retained if their discrimination is adequate and their IRT information covers the targeted range of the trait; decisions will always consider difficulty, discrimination, information, and content relevance together [33,77]. Corrected item–total (item–rest) correlations should be ≥0.30 (≥0.20 is acceptable for content-critical items) [33]. For the attitude items, full use of the response scale, inter-item correlations between 0.15 and 0.85 within each subscale, and item–rest correlations ≥ 0.30 are expected. Missing and “don’t know” responses will be reported for each item. Floor and ceiling effects will be defined as >15% of respondents obtaining the minimum or maximum score of a scale [37].

**Structural validity.** The field sample will be split at random into a development half and a confirmation half. In the development half, exploratory factor analysis will be performed on the tetrachoric (knowledge) and polychoric (attitude) correlation matrices, using principal-axis or weighted least-squares extraction with oblique rotation, in line with current recommendations [78]. Sampling adequacy will require a Kaiser–Meyer–Olkin (KMO) value ≥ 0.70 and a significant Bartlett test. The number of factors will be decided by parallel analysis, supported by the scree plot and by interpretability, and items are expected to load ≥0.40 on their target factor without cross-loadings ≥0.30. In the confirmation half, confirmatory factor analysis with an estimator suitable for categorical and ordinal items (weighted least squares with mean and variance adjustment, WLSMV) will test the resulting structure. Acceptable fit is defined as CFI and TLI ≥ 0.90 (≥0.95 good), RMSEA ≤ 0.08 (≤0.06 good), and SRMR ≤ 0.08, interpreted together rather than as rigid cut-offs [79]. The knowledge scale is expected to be essentially unidimensional. For the attitude items, the pre-specified two-factor model (perceived threat; perceived benefits and self-efficacy) will be compared with a one-factor model. The intention items and the formative practice index will not be included in the factor analyses. If fewer than 225 valid questionnaires are available per half or if the split-half solutions are unstable (non-convergence, improper estimates, or loadings that differ markedly between halves), confirmatory factor analysis will be performed on the full sample, and the exploratory solution will be checked by bootstrap resampling; any such change will be reported as a deviation from the protocol.

**Item response theory.** Complementing the classical analyses, the knowledge items and the attitude-subscale items will be calibrated on the pooled sample, with the final model selected by formal comparison rather than assumed [41,42,73]. For the dichotomously scored knowledge items, the one-parameter (1PL/Rasch), two-parameter (2PL), three-parameter (3PL, adding a lower asymptote for guessing, which is plausible with true/false statements), and four-parameter (4PL, adding an upper asymptote) logistic models will be compared; for the ordinal attitude items, the graded response model will be compared with the generalized partial credit model and the more restrictive partial credit and rating scale models. Nested models will be compared with likelihood-ratio tests, and all models with the Akaike and Bayesian information criteria (including the sample-size-adjusted BIC); because 3PL and 4PL parameters are unstable in samples of several hundred respondents, these models will be estimated with weakly informative priors on the asymptote parameters and retained only if they improve fit substantially and yield interpretable estimates, with parsimony being preferred when criteria disagree. Item discrimination and difficulty (or threshold) parameters, item and test information functions, overall model fit (M2 statistic and its RMSEA), item-level fit (S-*χ^2^*), and local independence (Q3 residual correlations) will be reported. Differential item functioning (DIF) will be screened across sex, age group (<50 vs. ≥50 years), educational level, and administration mode using IRT-based likelihood-ratio tests and ordinal logistic regression approaches; items with meaningful DIF will be flagged for revision or removal [80]. Because some subgroups will be relatively small, DIF results will be interpreted as screening findings, using effect-size criteria in addition to significance tests. The IRT results will inform final item selection alongside the classical criteria and will indicate whether the knowledge score covers the intended range of the latent trait.

**Reliability.** Internal consistency will be estimated with Kuder–Richardson 20 for the dichotomously scored knowledge items and Cronbach’s alpha for each attitude subscale, in each case complemented by McDonald’s omega based on the factor models, with 0.70–0.95 pre-specified as acceptable; omega will be reported alongside alpha because it relaxes the tau-equivalence assumption [81]. Test–retest reliability will be quantified by the intraclass correlation coefficient (two-way mixed-effects model, absolute agreement, single measurement) with ICC ≥ 0.70 required at scale level [37,82], and by quadratic-weighted kappa ≥ 0.60 at item level. Measurement error will be reported as the standard error of measurement and the smallest detectable change. Because the practice items form a formative index rather than a reflective scale, internal-consistency thresholds will not be enforced for it [48]; its stability will nevertheless be assessed by ICC, and its composition is justified by content validity. The stability of the single intention items will be evaluated by weighted kappa only.

**Construct validity (hypothesis testing).** A priori hypotheses, pre-specified before data analysis, include: (i) higher educational level is associated with higher knowledge scores (at least a small-to-moderate effect); (ii) participants with a personal or first-degree family history of VTE score higher on knowledge; (iii) the perceived benefits and self-efficacy subscale correlates positively with the practice index (expected *r* = 0.2–0.5); (iv) MEDAS and IPAQ indicators correlate positively, and FTND negatively, with the practice index; (v) participants who have heard of DVT/PE (screening item SC1) score systematically higher on knowledge; (vi) perceived threat correlates positively with the clinical/situational domain count of the burden index (expected *r* ≥ 0.10); and (vii) participants with a healthcare background score higher on knowledge. Construct validity will be considered supported if ≥75% of the a priori hypotheses are confirmed [37]. Measurement invariance of the factor models across sex, age group (<50 vs. ≥50 years), and administration mode (paper vs. online) will be tested by multi-group CFA at configural, metric, and scalar levels, with invariance accepted when ΔCFI ≤ 0.010 and ΔRMSEA ≤ 0.015 between nested models, following current tutorial guidance on transparent invariance testing [83]. If a subgroup contains fewer than 150 participants, invariance testing for that variable will be reported as exploratory.

**Item reduction and finalization.** Items failing pre-specified criteria will be reviewed jointly against their statistical performance (classical and IRT) and content relevance; deletion decisions require both statistical redundancy or malfunction and expert agreement that content coverage is preserved. Using the IRT information functions, a short form of the knowledge scale (approximately eight items) will additionally be derived and its agreement with the full scale reported, to support administration where respondent burden must be minimized. The final instrument, its factor structure, and all measurement properties will be reported in full, following COSMIN reporting standards [32,34], so that the questionnaire can be reused and re-validated in other Romanian-speaking populations.

### 2.9. Data Management and Bias

Data will be stored in a password-protected, access-restricted database; only pseudonymized data will be analyzed. Selection bias inherent to convenience and online sampling will be mitigated by combining recruitment channels, by predefined quotas in the in-person arm, and by reporting the sociodemographic distribution against regional census data; the potential effect will be examined in the weighting and subgroup sensitivity analyses described in Section 2.7. Question-order effects will be tested experimentally in the online arm (Section 2.6). Self-report and recall bias are acknowledged limitations of anthropometric and lifestyle measures; the balanced true/false design of the knowledge scale and neutral item wording limit acquiescence and social-desirability effects, which are discussed further in Section Limitations, Anticipated Risks, and Their Management.

### 2.10. Ethical Considerations

The study will be conducted in accordance with the Declaration of Helsinki and will begin only after approval by the institutional Research Ethics Committee. Participation is voluntary and anonymous; written or electronic informed consent is obtained before enrollment, separately covering the optional test–retest re-contact. Participants in the concept-elicitation interviews provide separate written consent for audio recording, and recordings are deleted after verified transcription. All data are processed in compliance with Regulation (EU) 2016/679 (GDPR). Because the risk-factor burden index is exploratory and does not estimate VTE probability, no individual risk feedback with clinical claims will be provided; participants receive general educational information on VTE prevention and are advised to consult a physician for any health concern.

## 3. Expected Results

As this article is a study protocol, no results are reported. This section describes the outputs anticipated from each phase.

Phase 1 is expected to yield a content-valid, comprehensible pre-final instrument whose content reflects concepts raised by lay participants (S-CVI/Ave ≥ 0.90, all I-CVI ≥ 0.78, feasibility criteria met). Phase 2 is expected to enroll at least 450 participants (target ≈ 600) and to establish the measurement properties of the PE-KAP-RO scales against the criteria in Table 4, including an essentially unidimensional, internally consistent knowledge scale with adequate IRT item fit and no meaningful DIF, a two-factor attitude structure (perceived threat; perceived benefits and self-efficacy) with adequate model fit, satisfactory test–retest reliability, and confirmation of at least 75% of the a priori construct-validity hypotheses; a limited number of items may be revised or removed in the finalization step. Substantively and in line with the awareness gaps reported internationally [22,23,24], we anticipate identifying deficits in the recognition of PE warning signs and of the roles of immobilization, obesity, diet, and smoking; higher educational level, younger age, and healthier lifestyle indicators are expected to be associated with higher knowledge and more favorable practices. The prevalence of individual self-reported risk factors and the distribution of the two domain counts of the exploratory burden index will be described, together with their associations with KAP dimensions; these descriptive results can inform, but not replace, a future, separately designed predictive study.

## 4. Discussion

This study will provide, to our knowledge, the first psychometrically validated instrument for assessing PE-related knowledge, attitudes, and practices among adults in Romania, developed and evaluated according to COSMIN-based standards rather than used ad hoc [32,33,34]. Its strengths include a theory-anchored attitude scale, a knowledge scale designed against acquiescence bias, formal content validation, a pre-specified split-sample factor-analytic strategy complemented by item response theory and DIF screening, per-analysis sample-size justification, test–retest assessment, integration of validated lifestyle instruments (IPAQ-SF, MEDAS, FTND) rarely combined in this context [49,52,55], and dual recruitment with formal invariance testing across administration modes. A validated instrument fills a concrete measurement gap: existing surveys of VTE awareness, although consistent in demonstrating low knowledge, have relied on unvalidated questionnaires that preclude comparison across settings and over time [22,23,24,25], and the impact of international awareness initiatives cannot be evaluated without standardized measures [84]. In a country where limited health literacy is common [85] and health literacy is linked to cardiovascular outcomes [86], such measurement is a precondition for effective, evaluable education—especially since structured educational interventions have been shown to change thromboprophylaxis-related behavior [87].

A reasonable alternative would have been to translate and validate an existing questionnaire for use in Romania. We did not identify an instrument matching the intended construct: questionnaires with documented measurement properties in this field address other populations or constructs—VTE-prevention knowledge and practice among nurses [29] or anticoagulation knowledge among treated patients [30]—whereas surveys of public awareness, including the multinational survey conducted for World Thrombosis Day [31] and recent KAP studies [22,23,24], used descriptive questionnaires whose measurement properties were not formally evaluated and which do not cover lifestyle-related risk factors or preventive practices in the general population. Cross-cultural validation of any of these would therefore have measured a different construct or required changes extensive enough to amount to new instrument development. Where suitable validated instruments exist—for physical activity, diet, and nicotine dependence—they are embedded and cross-culturally adapted rather than replaced.

Respondent burden is a legitimate concern for a multi-module instrument. The purpose-built KAP core (one screening item and 34 KAP items) can be administered on its own, whereas the lifestyle modules (IPAQ-SF, MEDAS, FTND, alcohol) are embedded to support construct validation and can be omitted where only KAP outcomes are required; the FTND applies only to current smokers, and the online version uses skip logic. The pilot will verify that the median completion time does not exceed 15 min, and the finalization step will derive an IRT-based short knowledge form (Section 2.8). Prompt recognition of warning signs and timely care-seeking, captured by item AT11 and the knowledge items on symptoms, are relevant because delays between symptom onset, diagnosis, and treatment carry prognostic consequences, as documented for other serious conditions in Western Romania [88]. Beyond research, a validated instrument could be used to evaluate educational campaigns, for which the increased public attention to thrombotic disease since the COVID-19 pandemic—accompanied by an excess of venous thromboembolic events and measurable changes in PE-related mortality, including in Romania [89,90,91]—offers a timely opportunity.

The risk-factor module is deliberately summarized by an exploratory, unweighted burden index reported in two separate domains rather than by a weighted score. Once the definitions and weights of established models are modified and lifestyle variables are added, the result is a new instrument whose weights have no empirical basis in the target population, and a single total could suggest that several modest lifestyle factors are clinically equivalent to a previous VTE or active cancer [35,61,62]. The present study therefore analyzes risk factors individually and uses the domain counts descriptively only.

The findings will constitute the population-level component (Direction 1) of a broader doctoral research program on prognostic elements in pulmonary embolism. The subsequent directions of the program—a prospective observational cohort of patients with confirmed PE, in which established prognostic instruments such as the PESI and sPESI are applied [67,68], and an artificial-intelligence-assisted analysis of computed tomography pulmonary angiography [92,93]—address prognosis after PE has occurred and are not designed to validate the burden index: the PESI and sPESI predict outcomes in patients with established PE rather than the risk of developing VTE, and a cohort restricted to patients with PE cannot assess discrimination between people who will and will not develop the disease. At most, comparing risk-factor frequencies between patients with PE and the community sample would be hypothesis-generating. Should a community risk tool be pursued, it will require a dedicated cohort of adults at risk followed for incident, objectively confirmed VTE, with derivation and validation according to current prediction-model standards [39,65,66]. The validated PE-KAP-RO instrument itself is intended to serve as a reusable outcome measure for educational campaigns and preventive interventions in Romanian-speaking populations.

### Limitations, Anticipated Risks, and Their Management

The main limitations of the planned study, the risks they pose for the interpretation of the findings, and the strategies pre-specified to manage them are summarized in Table 5. The most important concerns the size of the sample relative to the analytical plan. The planned sample (at least 450 valid questionnaires, target ≈ 600) meets commonly cited benchmarks for the descriptive aims and for factor analysis, but split-sample factor analysis and IRT are demanding. Simulation studies show that the sample needed for factor analysis depends on the communalities of the items and on how well each factor is determined by its indicators, rather than on fixed respondent-to-item ratios [94]. Monte Carlo studies of applied structural equation models likewise show that requirements depend on the number of indicators and factors and on the magnitude of the factor loadings, range widely (from about 30 to 460 cases), and are poorly captured by commonly cited rules of thumb [95]; the same Monte Carlo approach can be used to determine the sample size and power needed for a specific model before data collection [96]. Similarly, the accuracy of item parameter estimates in graded response models depends on the sample size and test length, with approximately 500 respondents providing accurate estimates in most simulated conditions [97]. With at least 225 participants per half, the factor analyses are expected to be adequate if loadings are at least moderate, whereas the DIF and invariance analyses, which compare smaller subgroups, have limited power. These analyses will therefore be interpreted as screening analyses, and the fallback strategies in Table 5 have been specified in advance, so that any change to the planned analysis is transparent and reported as a protocol deviation.

Further limitations follow from the design. The cross-sectional design precludes causal inference and predictive validation. Anthropometric, clinical, and lifestyle data are self-reported. Scoring “don’t know” responses as incorrect conservatively treats uncertainty as error. The measurement properties of the Romanian versions of the IPAQ-SF, MEDAS, and FTND will be established within this study rather than beforehand. Finally, quotas, census comparison, and weighting reduce but cannot remove selection bias: respondents reached online or through healthcare-related channels may differ in education, health awareness, digital literacy, or interest in cardiovascular disease. Estimates will therefore be presented as estimates for adults in Western Romania who can be reached through these channels, not as population prevalences.

## 5. Conclusions

This protocol defines a rigorous, two-phase study to develop and validate PE-KAP-RO, an integrated questionnaire assessing knowledge, attitudes, and practices regarding pulmonary embolism and its modifiable risk factors, together with a descriptive profile of self-reported risk factors, in adults from Western Romania. By pre-specifying the full psychometric evaluation—concept elicitation, content validity, item analysis, structural validity, item response theory calibration with model comparison and DIF screening, internal consistency, test–retest reliability, measurement error, construct validity, and measurement invariance—together with per-analysis sample-size justification, before participant enrollment, the study is designed to deliver a validated, reusable assessment tool rather than a single-use survey. The resulting evidence will quantify PE-related awareness, identify subgroups in need of targeted prevention, and provide a standardized outcome measure for future educational and preventive interventions.

## Figures and Tables

**Table 1:** Sample-size justification by planned analysis.

Analysis	Assumptions	Required *n*	Provision
Prevalence of adequate knowledge	*p* = 0.50 (most conservative), ±5%, 95% CI; +15% invalid questionnaires	384 (450 enrolled)	≥450, toward 600
KAP–lifestyle correlations	|*r*| ≥ 0.15, two-sided *α* = 0.05, power 0.80	≈347	≥450
Multivariable linear regression	Incremental *f^2^* = 0.02 per predictor, ≤15 predictors, power 0.80	≈395	≥450
Logistic regression (adequate knowledge)	Events per variable ≥ 10, ≤12 predictors, prevalence ≥ 30%	≥120 events	≈135 events at *n* = 450
EFA/CFA (random split halves)	≥7 × items and ≥100 per half; ≥10 respondents per item	105–150 per half	≥225 per half; Monte Carlo check before field testing
IRT calibration (model comparison)	Several hundred respondents; ≥500 desirable for stable 2PL/GRM estimates; 3PL/4PL with priors	≥450 (500+ desirable)	Pooled sample; recruitment toward 600; 3PL/4PL exploratory
DIF and measurement invariance	Subgroup comparisons (sex, age group, education, administration mode)	≈150 per group	Groups < 150: reported as exploratory
Test–retest reliability (ICC)	True ICC ≥ 0.80; 95% CI half-width ≤ 0.10; two administrations	≈50 pairs	≥60 invited
Order-effect test (online arm, randomized 1:1)	Standardized difference |*g*| ≥ 0.35 in attitude/practice scores, two-sided *α* = 0.05, power 0.80	≈130 per order condition	Online arm; minimum detectable effect reported
Concept elicitation (qualitative)	Purposive variation; recruitment until saturation	≈15–20	Until saturation
Pilot (face validity/feasibility)	Cognitive debriefing; clarity and timing criteria	≈ 30	30

CI—confidence interval; EFA/CFA—exploratory/confirmatory factor analysis; IRT—item response theory; 2PL/3PL/4PL—two-/three-/four-parameter logistic models; GRM—graded response model; DIF—differential item functioning; ICC—intraclass correlation coefficient. The binding requirement (enrollment ≥ 450) derives from the precision of the primary proportion estimate; all other requirements are met or exceeded at that size.

**Table 4:** Pre-specified measurement properties, methods, and acceptance criteria for the psychometric evaluation of PE-KAP-RO.

Measurement Property	Method	Acceptance Criterion
Content validity	Concept elicitation (*n* ≈ 15–20); expert panel (*n* ≥ 7), 4-point relevance ratings; I-CVI, S-CVI/Ave	All key elicited concepts covered; I-CVI ≥ 0.78 per item; S-CVI/Ave ≥ 0.90
Face validity/feasibility	Pilot (*n* ≈ 30), cognitive debriefing, clarity and relevance ratings, timing	≥80% rate each item clear; median completion ≤ 15 min; item missingness < 5%
Item difficulty (knowledge)	Proportion of correct answers per item	0.20–0.80 screening range; items outside (≤0.90 if content-critical) retained if discrimination and information are adequate
Item discrimination	Corrected item–total (item-rest) correlation	≥0.30 (≥0.20 acceptable for content-critical items)
Structural validity	Split sample: EFA (tetrachoric/polychoric, parallel analysis) → CFA (WLSMV)	KMO ≥ 0.70; loadings ≥ 0.40, cross-loadings < 0.30; CFI/TLI ≥ 0.90; RMSEA ≤ 0.08; SRMR ≤ 0.08
Item calibration (IRT)	Model comparison (1PL/2PL/3PL/4PL; RSM/PCM/GPCM/GRM) by LRT, AIC, BIC; M2, S-*χ^2^*, Q3 residuals	Adequate item fit (S-*χ^2^* *p* > 0.01); |Q3| < 0.20; information covering the trait range
Differential item functioning	IRT likelihood-ratio tests; ordinal logistic regression (sex, age, education, mode)	No meaningful DIF; flagged items revised or removed
Internal consistency	KR-20 (knowledge), Cronbach’s α (attitude subscales), McDonald’s ω (model-based)	0.70–0.95 (reflective scales only)
Test–retest reliability	ICC (two-way mixed, absolute agreement, single measure), 14 ± 4 days, *n* ≥ 50; item-level quadratic-weighted κ	ICC ≥ 0.70 (scale level); κ ≥ 0.60 (item level)
Measurement error	Standard error of measurement; smallest detectable change	Reported with 95% CI
Floor/ceiling effects	Percentage at minimum/maximum scale score	<15%
Construct validity	A priori hypothesis testing (known groups; convergent correlations)	≥75% of pre-specified hypotheses confirmed
Question-order effect	Randomized comparison in the online arm (standard vs. knowledge-first order)	|*g*| < 0.20 between order conditions
Measurement invariance	Multi-group CFA: sex; age < 50 vs. ≥50; paper vs. online	ΔCFI ≤ 0.010 and ΔRMSEA ≤ 0.015 (configural → metric → scalar)

EFA—exploratory factor analysis; CFA—confirmatory factor analysis; WLSMV—weighted least squares with mean and variance adjustment; KMO—Kaiser–Meyer–Olkin; CFI—comparative fit index; TLI—Tucker–Lewis index; RMSEA—root-mean-square error of approximation; SRMR—standardized root-mean-square residual; KR-20—Kuder–Richardson formula 20; ICC—intraclass correlation coefficient; CVI—content validity index; IRT—item response theory; 1PL/2PL/3PL/4PL—one-/two-/three-/four-parameter logistic models; RSM—rating scale model; PCM—partial credit model; GPCM—generalized partial credit model; GRM—graded response model; LRT—likelihood-ratio test; AIC/BIC—Akaike/Bayesian information criteria; DIF—differential item functioning.

**Table 5:** Anticipated risks, potential consequences, and planned management strategies. The main methodological and practical risks identified before enrollment are listed together with the strategies pre-specified to prevent or manage them.

Anticipated Risk	Potential Consequence	Planned Management
Recruitment below target (<450 valid questionnaires)	Lower precision; smaller split halves for EFA and CFA	Weekly monitoring of quotas and of the online arm; recruitment extended until ≥450 valid questionnaires; if the target is not reached, CFA on the full sample with bootstrap-checked EFA; achieved precision reported
Weak loadings or unstable factor structure	Non-convergence, improper solutions, or poor model fit	Monte Carlo simulation before field testing; full-sample CFA with bootstrap validation if split halves are unstable; items revised only with expert agreement; deviations reported
Very easy or very difficult knowledge items	Sparse response categories; unstable tetrachoric correlations and IRT estimates	Difficulty screening (Section 2.8); robust WLSMV estimation; preference for the simpler 1PL/2PL models
Instability of complex IRT models (3PL/4PL)	Biased or uninterpretable asymptote parameters	Weakly informative priors; retained only with a clear gain in fit; otherwise 2PL or GRM
Small subgroups for DIF and invariance testing	Low power; false-negative or false-positive findings	Effect-size criteria alongside significance tests; categories collapsed where justified (e.g., education); groups < 150 reported as exploratory
Attrition in the test–retest subsample	Fewer than 50 pairs; wide confidence intervals for the ICC	At least 60 participants invited; reminders through the preferred channel; precision of the ICC reported
Selection bias from non-probability sampling	Limited generalizability of descriptive estimates	Demographic quotas; comparison with census data; raking weights; results presented as sample estimates
Question-order and cueing effects	Biased attitude and practice responses	Attitudes and practices placed before knowledge; randomized order test in the online arm; sensitivity analysis
Self-report, recall, and social-desirability bias	Misclassification of BMI, lifestyle, and practices	Anonymous completion; neutral wording; balanced true/false statements; acknowledged in interpretation
Careless or duplicate online responses	Invalid data	Completion-time, straight-lining, and consistency checks; one submission per device; exclusions documented
Romanian versions of the IPAQ-SF, MEDAS, and FTND without published validation	Uncertain measurement properties of these modules	Cross-cultural adaptation following established guidelines; test–retest reliability and FTND internal consistency reported
Respondent burden and missing data	Incomplete questionnaires; lower data quality	Completion time verified in the pilot (≤15 min); skip logic online; multiple imputation as a sensitivity analysis
Cross-sectional design	No causal or predictive inference	Associational wording; burden index used descriptively only

EFA/CFA—exploratory/confirmatory factor analysis; IRT—item response theory; 1PL/2PL/3PL/4PL—one-/two-/three-/four-parameter logistic models; GRM—graded response model; DIF—differential item functioning; WLSMV—weighted least squares with mean and variance adjustment; ICC—intraclass correlation coefficient; BMI—body-mass index; IPAQ-SF—International Physical Activity Questionnaire–Short Form; MEDAS—Mediterranean Diet Adherence Screener; FTND—Fagerström Test for Nicotine Dependence.

## Data Availability

The datasets generated during the study will be available from the corresponding author on reasonable request, subject to ethical and data-protection constraints. The full study instrument is provided in Appendix A and Appendix B.

## Supplementary Materials

The following supporting information can be downloaded at: https://www.mdpi.com/article/10.3390/healthcare14213599/s1, Supplementary File S1: The original Romanian version of the PE-KAP-RO questionnaire as administered to participants (cited in Section 2.4.1 and Section 2.4.4); Supplementary File S2: English translation of the participant form (also reproduced in Appendix A).

## Appendix A. The PE-KAP-RO Questionnaire (English Version)

The instrument is administered in Romanian, with sections in the order shown; the English version below was obtained by forward–backward translation and is provided for transparency and reuse. Investigator-scored fields are marked “(investigator)”. Answer keys for the knowledge scale (T = true, F = false) and all scoring rules are given in Appendix B and do not appear on the participant form.

Participant information (header of the form): Thank you for taking part. Completion is voluntary and anonymous and takes approximately 15 min. There are no “right” or “wrong” answers to the questions about attitudes and habits—please answer honestly. Data are used exclusively for research, processed in aggregate, in accordance with Regulation (EU) 2016/679 (GDPR).

*Section* *A. Sociodemographic and Anthropometric Data*

Participant code (investigator): ________ Date of completion: ____/____/________

A1. Age (completed years): ________

A2. Sex:

☐ Female ☐ Male

A3. Area of residence:

☐ Urban ☐ Rural

A4. Educational level:

☐ No formal/primary education ☐ Lower secondary ☐ Upper secondary/post-secondary ☐ University/postgraduate

A5. Occupational status/type of activity:

☐ Predominantly sedentary (desk) work ☐ Predominantly physical work ☐ Unemployed ☐ Retired ☐ Student ☐ Homemaker

A6. Height: ______ cm Current weight: ______ kg BMI (investigator, kg/m^2^): ______

A7. Waist circumference (if known): ______ cm

A8. Do you work or study in the healthcare field?

☐ No ☐ Yes—physician ☐ Yes—nurse, midwife, or other health professional ☐ Yes—pharmacist ☐ Yes—student in a health-related field

*Section B. Personal History and Risk Factors*

Please tick what applies to you. These data are self-reported.

B1. Have you ever been diagnosed with venous thrombosis (DVT) or pulmonary embolism (PE)?

☐ Yes ☐ No ☐ Don’t know

B2. Has a first-degree relative (parent, sibling, child) had venous thrombosis/PE?

☐ Yes ☐ No ☐ Don’t know

B3. Do you have a known thrombophilia (a diagnosed clotting disorder)?

☐ Yes ☐ No ☐ Don’t know

B4. Cancer (active, or treated within the last 5 years)?

☐ Yes ☐ No

B5. Major surgery, or trauma/fracture, in the last 3 months?

☐ Yes ☐ No

B6. Prolonged immobilization or hospitalization (≥3 days in bed) in the last 3 months?

☐ Yes ☐ No

B7. Long-distance travel (>4 h, by plane or car) in the last month?

☐ Yes ☐ No

B8. Women only—hormonal therapy/pregnancy:

☐ Hormonal contraceptives ☐ Hormone replacement therapy ☐ Pregnancy/postpartum (last 6 weeks) ☐ None/not applicable

B9. Known chronic conditions (tick all that apply):

☐ Hypertension ☐ Diabetes mellitus ☐ Dyslipidemia ☐ Heart disease ☐ Chronic respiratory disease ☐ Chronic kidney disease ☐ Varicose veins/chronic venous insufficiency ☐ None

B10. Are you currently taking anticoagulant or antiplatelet treatment?

☐ Yes ☐ No ☐ Don’t know

*Section C. Awareness and Attitudes*

SC1. Have you ever heard of deep vein thrombosis (DVT) or pulmonary embolism (PE)?

☐ Yes ☐ No

Please indicate how much you agree with each statement (1 = strongly disagree … 5 = strongly agree).

Statement	1 Strongly Disagree	2	3 Neutral	4	5 Strongly Agree
AT1. I consider thrombosis/pulmonary embolism to be a serious condition.	○	○	○	○	○
AT2. If I developed a blood clot, it would seriously affect my health and daily life.	○	○	○	○	○
AT3. I believe that I personally could be at risk of thrombosis.	○	○	○	○	○
AT4. Given my health and lifestyle, it is possible that I will develop a blood clot at some point in my life.	○	○	○	○	○
AT5. Preventing thrombosis is important for my health.	○	○	○	○	○
AT6. Taking preventive measures in daily life would lower my chance of developing a blood clot.	○	○	○	○	○
AT7. I feel able to take concrete preventive measures in my daily life.	○	○	○	○	○
AT8. I am confident that I could change my daily habits if a doctor told me I was at risk of thrombosis.	○	○	○	○	○
AT9. I am willing to change my lifestyle to reduce my risk of thrombosis.	○	○	○	○	○
AT10. I would like to receive more information about preventing thrombosis.	○	○	○	○	○
AT11. If I noticed symptoms that could indicate a blood clot, I would seek medical care quickly.	○	○	○	○	○

*Section D. Preventive Practices and Behaviors*

PR1. When you sit for a long time (at work, at home, in front of a screen), how often do you get up and move around at least once every hour?

☐ Usually ☐ Sometimes ☐ Rarely or never

PR2. When you have to stay seated for a long time, do you move or flex your feet and legs?

☐ Usually ☐ Sometimes ☐ Rarely or never

PR3. Do you monitor your body weight?

☐ Yes, regularly ☐ Occasionally ☐ No

PR4. How often do you see a doctor for a check-up?

☐ At least once a year ☐ Less than once a year ☐ Never

PR5. Have you ever looked for information about preventing blood clots (doctor, leaflets, internet)?

☐ Several times ☐ Once or twice ☐ Never

PR6. Have you ever talked with a doctor, nurse, or pharmacist about your own risk of blood clots (for example, when starting a hormonal treatment, before surgery, or at a check-up)?

☐ Yes, more than once ☐ Yes, once ☐ No

The next two questions apply only to some situations; tick “not applicable” if the situation does not apply to you.

PR7. On long journeys (>4 h), do you usually get up/stop and move around at regular intervals?

☐ Always ☐ Sometimes ☐ Never ☐ Not applicable (I do not make long journeys)

PR8. When prevention of thrombosis was recommended during a previous hospitalization or operation (injections and/or compression stockings), did you follow it?

☐ Yes, completely ☐ Only partly ☐ No, I did not follow it ☐ Not applicable (never hospitalized/never recommended)

*Section E. Knowledge About Thrombosis and Pulmonary Embolism*

For each statement, mark whether you consider it true or false, or tick “Don’t know”.

Statement	True	False	Don’t Know
K1. A pulmonary embolism is a blood clot that blocks the arteries of the lungs.	○	○	○
K2. Deep vein thrombosis most often develops in the veins of the legs.	○	○	○
K3. Prolonged immobilization (bed rest, sitting still for long periods) increases the risk of blood clots.	○	○	○
K4. Only elderly people can develop venous thrombosis.	○	○	○
K5. Obesity increases the risk of thrombosis.	○	○	○
K6. Smoking has no influence on the risk of blood clots.	○	○	○
K7. Long journeys (over 4 h) by plane or car can increase the risk of blood clots.	○	○	○
K8. Hormonal contraceptives can increase the risk of thrombosis in women.	○	○	○
K9. Cancer has no effect on the risk of blood clots.	○	○	○
K10. Pregnancy and the first weeks after childbirth increase the risk of thrombosis.	○	○	○
K11. Pain and sudden swelling of one calf can be signs of deep vein thrombosis.	○	○	○
K12. Sudden shortness of breath and chest pain can be signs of pulmonary embolism.	○	○	○
K13. Regular physical activity increases the risk of thrombosis.	○	○	○
K14. A diet high in red and processed meat may be associated with a higher risk of thrombosis.	○	○	○
K15. A pulmonary embolism is never life-threatening.	○	○	○

Knowledge score (investigator): 1 point per correct answer; “don’t know” = 0 points (total 0–15).

*Section F. Physical Activity—International Physical Activity Questionnaire, Short Form (IPAQ-SF)*

The questions refer to time spent in physical activity during the last 7 days. Count only activities performed for at least 10 min without interruption.

F1. During the last 7 days, on how many days did you do VIGOROUS physical activities (hard effort, much faster breathing—e.g., heavy lifting, digging, running, aerobics, fast cycling)?

days per week: ______ or ☐ none → go to question F3

F2. On those days, how much time did you usually spend per day on vigorous activities?

hours: ______ minutes: ______

F3. During the last 7 days, on how many days did you do MODERATE physical activities (moderate effort, somewhat faster breathing—e.g., carrying light loads, cycling at a regular pace)? Do NOT include walking.

days per week: ______ or ☐ none → go to question F5

F4. On those days, how much time did you usually spend per day on moderate activities?

hours: ______ minutes: ______

F5. During the last 7 days, on how many days did you WALK for at least 10 min at a time (at work, at home, for transport, sport, or leisure)?

days per week: ______ or ☐ none → go to question F7

F6. On those days, how much time did you usually spend walking per day?

hours: ______ minutes: ______

F7. On a usual weekday, how much time did you spend SITTING (desk, table, TV, transport)?

hours: ______ minutes: ______

Scoring (investigator): MET-min/week = walking 3.3 × min × days + moderate 4.0 × min × days + vigorous 8.0 × min × days. Data processing: bouts <10 min excluded; each activity type truncated at 180 min/day; cases with a total >960 min/day excluded. Categories: High (vigorous activity on ≥3 days accumulating ≥1500 MET-min/week; OR ≥ 7 days of any combination of walking, moderate, or vigorous activity accumulating ≥3000 MET-min/week)/Moderate (not High, and ≥3 days of vigorous activity ≥20 min/day; OR ≥ 5 days of moderate activity and/or walking ≥30 min/day; OR ≥ 5 days of any combination accumulating ≥600 MET-min/week)/Low (neither criterion met).

*Section G. Dietary Habits—Mediterranean Diet Adherence Screener (MEDAS, 14 Items)*

Please think of your usual eating habits over the recent period. Tick the option that best describes your habit. The option marked “(1p)” receives 1 point.

G1. Do you use olive oil as your main fat for cooking?

☐ Yes (1p) ☐ No

G2. How much olive oil do you consume in total per day (cooking, salads, meals away from home)? (1 tablespoon ≈ 10 mL)

☐ ≥4 tablespoons (1p) ☐ <4 tablespoons

G3. How many servings of vegetables do you consume per day? (1 serving = 200 g; a side dish = ½ serving)

☐ ≥2 servings, of which ≥1 raw or as salad (1p) ☐ <2 servings

G4. How many pieces of fruit (including natural fruit juice) do you consume per day?

☐ ≥3 (1p) ☐ <3

G5. How many servings of red meat, hamburger, or processed meat do you consume per day? (1 serving = 100–150 g)

☐ <1 (1p) ☐ ≥1

G6. How many servings of butter, margarine, or cream do you consume per day? (1 serving = 12 g)

☐ <1 (1p) ☐ ≥1

G7. How many sweetened or carbonated beverages do you drink per day?

☐ <1 (1p) ☐ ≥1

G8. How much wine do you drink per week? (Original MEDAS item; alcohol intake is also recorded separately in Section I and interpreted with caution.)

☐ ≥7 glasses (1p) ☐ <7 glasses

G9. How many servings of legumes (beans, peas, lentils, chickpeas) do you consume per week? (1 serving = 150 g)

☐ ≥3 (1p) ☐ <3

G10. How many servings of fish or seafood do you consume per week? (100–150 g fish/200 g seafood)

☐ ≥3 (1p) ☐ <3

G11. How many times per week do you consume commercial sweets or pastries (cakes, biscuits, sponge cakes)?

☐ <3 (1p) ☐ ≥3

G12. How many servings of nuts (including peanuts) do you consume per week? (1 serving = 30 g)

☐ ≥3 (1p) ☐ <3

G13. Do you prefer chicken, turkey, or rabbit instead of beef, pork, hamburger, or sausages?

☐ Yes (1p) ☐ No

G14. How many times per week do you consume vegetables, pasta, rice, or other dishes prepared with a “sofrito” sauce (tomatoes with onion/garlic/leek simmered in olive oil)?

☐ ≥2 (1p) ☐ <2

Total MEDAS score (investigator, 0–14): ______ | Interpretation: ≤5 low adherence; 6–9 moderate; ≥10 high adherence.

*Section H. Smoking—Status and Fagerström Test for Nicotine Dependence (FTND)*

H1. What is your smoking status?

☐ Never smoker ☐ Former smoker ☐ Current smoker

If former smoker, how many years since you quit? ______ years

Number of cigarettes per day (currently, or during the smoking period): ______ cigarettes/day

Total years of smoking: ______ years → Pack-years (investigator: cigarettes/day ÷ 20 × years): ______

The FTND questions below are completed ONLY by current smokers. Tick one answer; the score is shown in parentheses.

H2.1. How soon after waking do you smoke your first cigarette?

☐ Within 5 min (3) ☐ 6–30 min (2) ☐ 31–60 min (1) ☐ After 60 min (0)

H2.2. Do you find it difficult to refrain from smoking in places where it is forbidden (church, cinema, train, hospital)?

☐ Yes (1) ☐ No (0)

H2.3. Which cigarette would you hate most to give up?

☐ The first one in the morning (1) ☐ Any other (0)

H2.4. How many cigarettes do you smoke per day?

☐ ≤10 (0) ☐ 11–20 (1) ☐ 21–30 (2) ☐ ≥31 (3)

H2.5. Do you smoke more frequently during the first hours after waking than during the rest of the day?

☐ Yes (1) ☐ No (0)

H2.6. Do you smoke even when you are so ill that you are in bed most of the day?

☐ Yes (1) ☐ No (0)

Total FTND score (investigator, 0–10): ______ | Interpretation: 0–2 very low; 3–4 low; 5 moderate; 6–7 high; 8–10 very high dependence.

*Section I. Alcohol Intake*

I1. Do you drink alcoholic beverages?

☐ No, never ☐ Yes

I2. How often do you drink alcohol?

☐ Occasionally (<1/week) ☐ 1–3 times/week ☐ 4–6 times/week ☐ Daily

I3. Approximate number of units of alcohol per week: ______ units

1 unit ≈ 12 g pure alcohol ≈ 330 mL beer (5%) ≈ 125 mL wine (12%) ≈ 40 mL spirits (40%).

Thank you for your time!

## Appendix B. Scoring Algorithm and Provisional Interpretation

### Appendix B.1. Scale Scores

**Knowledge (0–15).** One point per correct answer; “don’t know” = 0. Answer key: true for K1–K3, K5, K7, K8, K10–K12, K14; false for K4, K6, K9, K13, K15. Provisional bands: <8 low, 8–11 moderate, ≥12 high; adequate knowledge = ≥11 points (≥70% correct).

**Attitudes.** Perceived threat subscale = sum of AT1–AT4 (4–20); perceived benefits and self-efficacy subscale = sum of AT5–AT8 (4–20). Provisional bands per subscale: ≥16 favorable (item mean ≥ 4), 12–15 neutral-to-positive, < 12 unfavorable. No total attitude score is computed. AT9 (willingness to change), AT10 (information-seeking), and AT11 (care-seeking intention) are reported individually (1–5; agreement = 4–5). Construct mapping: AT1–AT2 perceived severity; AT3–AT4 perceived susceptibility; AT5–AT6 perceived benefits; AT7–AT8 self-efficacy. The screening item SC1 is not scored.

**Practices (0–12).** Core items PR1–PR6 are scored 2/1/0 in the order the options are listed (PR1–PR2: usually 2, sometimes 1, rarely or never 0; PR3: regularly 2, occasionally 1, no 0; PR4: at least yearly 2, less often 1, never 0; PR5: several times 2, once or twice 1, never 0; PR6: more than once 2, once 1, no 0) and summed (0–12). If exactly one core item is missing, the total is prorated (mean of the five answered items × 6); with ≥2 missing, the index is not computed. The index is also expressed as a percentage: ≥75% good, 50–74% moderate, <50% limited preventive practice. PR7 (always 2, sometimes 1, never 0) and PR8 (completely 2, partly 1, not followed 0) are situation-specific, are not included in the index, and are reported descriptively among participants to whom they apply.

**Validated modules.** IPAQ-SF, MEDAS, and FTND are scored according to their original algorithms, as printed on the form (Section F–H): IPAQ-SF—official data-processing and categorical rules; MEDAS—≤5 low, 6–9 moderate, ≥10 high adherence; FTND—0–2 very low, 3–4 low, 5 moderate, 6–7 high, 8–10 very high dependence.

### Appendix B.2. Exploratory VTE Risk-Factor Burden Index

Computed from Section A, B, F, G, and H responses as two unweighted counts of the elements listed in Table 3 (main text): a clinical/situational domain count (0–10) and a lifestyle domain count (0–4). “Don’t know” counts as absent (0), and the proportion of “don’t know” responses is reported for each element. The two counts are reported separately and are not summed, and no risk categories are defined. The index describes self-reported risk-factor burden only: it does not estimate the probability of VTE, is not a diagnostic or prognostic instrument, and must not replace medical evaluation; participants receive only general preventive education and the advice to discuss risk factors with a physician.

All thresholds in this appendix are provisional and will be finalized after the psychometric evaluation described in Section 2.8.
