# Supplementary material for: Development and Psychometric Validation of a Knowledge, Attitudes, and Practices Questionnaire on Pulmonary Embolism and Its Modifiable Risk Factors (PE-KAP-RO): Protocol for a Cross-Sectional Study in Adults from Western Romania
Source: Healthcare (Basel). 2026 Oct 23;14(21):3599. doi: 10.3390/healthcare14213599 (PMC13762760; doi:10.3390/healthcare14213599)
Supplement: Supplementary file 1 [file healthcare-14-03599-s001.zip › healthcare-4553484_Supplementary_File_S1.pdf]

CHESTIONARUL PE-KAP-RO privind cunoștințele, atitudinile și practicile referitoare la trombembolismul pulmonar și factorii săi de risc

Studiu transversal de dezvoltare și validare la adulți din vestul României (Direcția 1)

Vă mulțumim pentru participare. Completarea chestionarului este voluntară și anonimă și durează aproximativ 15 minute. Nu există răspunsuri «corecte» sau «greșite» la întrebările despre atitudini și obiceiuri — vă rugăm să răspundeți sincer. Datele vor fi utilizate exclusiv în scop de cercetare, prelucrate agregat, conform Regulamentului (UE) 2016/679 (GDPR).

Consimțământ informat

Am citit și am înțeles informațiile de mai sus privind scopul studiului și modul de prelucrare a datelor. Înțeleg că participarea este voluntară și anonimă, că răspunsurile mele nu conțin date de identificare directă și că pot întrerupe completarea în orice moment, fără nicio consecință. Operator de date: Universitatea de Medicină și Farmacie «Victor Babeș» din Timișoara; prelucrarea se realizează în conformitate cu Regulamentul (UE) 2016/679 (GDPR).

☐ DA, sunt de acord să particip la acest studiu și ca datele furnizate de mine să fie utilizate în scop de cercetare științifică, inclusiv publicarea rezultatelor în formă agregată și anonimizată.

☐ DA, sunt de acord să fiu recontactat(ă) pentru a doua completare a chestionarului (test–retest, după aproximativ 14 zile) — opțional. Modalitate de contact (telefon/e-mail, opțional): ______________________
(datele de contact se stochează separat de răspunsuri și se șterg după a doua completare)

Chestionarele fără prima căsuță de consimțământ bifată nu vor fi prelucrate.

Secțiunea A. Date sociodemografice și antropometrice

Cod participant (investigator): ________ Data completării: ____ / ____ / ________

A1. Vârsta (ani împliniți): ________

A2. Sex:
☐ Feminin ☐ Masculin

A3. Mediu de proveniență:
☐ Urban ☐ Rural

A4. Nivel de educație:
☐ Fără studii / primar ☐ Gimnazial ☐ Liceal / postliceal ☐ Universitar / postuniversitar

A5. Statut ocupațional / tip de activitate:
☐ Muncă preponderent sedentară (birou) ☐ Muncă preponderent fizică ☐ Șomer ☐ Pensionar ☐ Student ☐ Casnic(ă)

A6. Înălțime: ______ cm Greutate (actuală): ______ kg IMC (investigator, kg/m²): ______

A7. Circumferință abdominală (dacă se cunoaște): ______ cm

A8. Lucrați sau studiați în domeniul sănătății?
☐ Nu ☐ Da — medic ☐ Da — asistent(ă) medical(ă), moașă sau alt profesionist din sănătate ☐ Da — farmacist ☐ Da — student(ă) într-un domeniu medical/sanitar

Secțiunea B. Antecedente și factori de risc

Bifați ceea ce vi se aplică. Aceste date sunt auto-raportate.

B1. Ați fost vreodată diagnosticat(ă) cu tromboză venoasă (TVP) sau trombembolism pulmonar (TEP)?
☐ Da ☐ Nu ☐ Nu știu

B2. O rudă de gradul I (părinte, frate/soră, copil) a avut tromboză venoasă / TEP?
☐ Da ☐ Nu ☐ Nu știu

B3. Aveți o trombofilie cunoscută (tulburare de coagulare diagnosticată)?
☐ Da ☐ Nu ☐ Nu știu

B4. Cancer (activ sau tratat în ultimii 5 ani)?
☐ Da ☐ Nu

B5. Intervenție chirurgicală majoră sau traumatism / fractură în ultimele 3 luni?
☐ Da ☐ Nu

B6. Imobilizare prelungită sau spitalizare (≥ 3 zile la pat) în ultimele 3 luni?
☐ Da ☐ Nu

B7. Călătorie lungă (> 4 ore, avion/mașină) în ultima lună?
☐ Da ☐ Nu

B8. Doar pentru femei — terapie hormonală / sarcină:
☐ Contraceptive hormonale ☐ Tratament hormonal de substituție ☐ Sarcină / lăuzie (ultimele 6 săpt.) ☐ Niciuna / nu se aplică

B9. Afecțiuni cronice cunoscute (bifați toate cele valabile):
☐ Hipertensiune arterială ☐ Diabet zaharat ☐ Dislipidemie ☐ Boală cardiacă ☐ Boală respiratorie cronică ☐ Boală renală cronică ☐ Varice / insuficiență venoasă ☐ Niciuna

B10. Urmați în prezent tratament anticoagulant sau antiagregant?

☐ Da ☐ Nu ☐ Nu știu

Secțiunea C. Conștientizare și atitudini

SC1. Ați auzit vreodată de tromboza venoasă profundă (TVP) sau de trombembolismul pulmonar (TEP)?

☐ Da ☐ Nu

Indicați în ce măsură sunteți de acord cu fiecare afirmație (1 = dezacord total … 5 = acord total).

Afirmație	1 Dezacord total	2	3 Neutru	4	5 Acord total
AT1. Consider că tromboza / trombembolismul pulmonar este o afecțiune gravă.	○	○	○	○	○
AT2. Dacă aș face un cheag de sânge, acesta mi-ar afecta serios sănătatea și viața de zi cu zi.	○	○	○	○	○
AT3. Cred că eu, personal, aș putea fi expus(ă) riscului de tromboză.	○	○	○	○	○
AT4. Având în vedere starea mea de sănătate și stilul meu de viață, este posibil să fac un cheag de sânge la un moment dat în viață.	○	○	○	○	○
AT5. Prevenirea trombozei este importantă pentru sănătatea mea.	○	○	○	○	○
AT6. Măsurile de prevenție din viața de zi cu zi mi-ar reduce șansa de a face un cheag de sânge.	○	○	○	○	○
AT7. Mă simt capabil(ă) să iau măsuri concrete de prevenție în viața de zi cu zi.	○	○	○	○	○
AT8. Sunt încrezător/încrezătoare că mi-aș putea schimba obiceiurile zilnice dacă un medic mi-ar spune că am risc de tromboză.	○	○	○	○	○
AT9. Sunt dispus(ă) să îmi modific stilul de viață pentru a-mi reduce riscul de tromboză.	○	○	○	○	○
AT10. Aș dori să primesc mai multe informații despre prevenirea trombozei.	○	○	○	○	○
AT11. Dacă aș observa simptome care ar putea indica un cheag de sânge, m-aș adresa rapid medicului.	○	○	○	○	○

Secțiunea D. Practici și comportamente preventive

PR1. Când stați mult timp așezat(ă) (la serviciu, acasă, în fața unui ecran), cât de des vă ridicați și vă mișcați cel puțin o dată pe oră?

☐ De obicei ☐ Uneori ☐ Rar sau niciodată

PR2. Când trebuie să stați mult timp așezat(ă), vă mișcați sau vă flexați picioarele și labele picioarelor?

☐ De obicei ☐ Uneori ☐ Rar sau niciodată

PR3. Vă monitorizați greutatea corporală?

☐ Da, regulat ☐ Ocazional ☐ Nu

PR4. Cât de des mergeți la medic pentru un control?

☐ Cel puțin o dată pe an ☐ Mai rar de o dată pe an ☐ Niciodată

PR5. Ați căutat vreodată informații despre prevenirea cheagurilor de sânge (medic, broșuri, internet)?

☐ De mai multe ori ☐ O dată sau de două ori ☐ Niciodată

PR6. Ați discutat vreodată cu un medic, o asistentă sau un farmacist despre propriul risc de cheaguri de sânge (de exemplu, la începerea unui tratament hormonal, înaintea unei operații sau la un control)?

☐ Da, de mai multe ori ☐ Da, o dată ☐ Nu

Următoarele două întrebări se aplică doar în anumite situații; bifați «Nu se aplică» dacă situația nu vă privește.

PR7. Când efectuați călătorii lungi (> 4 ore), obișnuiți să vă ridicați / să opriți și să vă mișcați periodic?

☐ Întotdeauna ☐ Uneori ☐ Niciodată ☐ Nu se aplică (nu efectuez călătorii lungi)

PR8. Când vi s-a recomandat profilaxia trombozei la o spitalizare sau operație anterioară (injecții și/sau ciorapi compresivi), ați urmat-o?

☐ Da, în totalitate ☐ Doar parțial ☐ Nu am urmat-o ☐ Nu se aplică (nu am fost spitalizat(ă) / nu mi s-a recomandat)

Secțiunea E. Cunoștințe despre tromboză și trombembolismul pulmonar

Pentru fiecare afirmație, marcați dacă o considerați adevărată sau falsă, ori bifați «Nu știu». Unele afirmații sunt adevărate, altele sunt false.

Afirmație	Adevărat	Fals	Nu știu
K1. Trombembolismul pulmonar este un cheag de sânge care blochează arterele plămânilor.	○	○	○
K2. Tromboza venoasă profundă apare cel mai frecvent la nivelul venelor picioarelor.	○	○	○
K3. Imobilizarea prelungită (repaus la pat, ședere îndelungată) crește riscul de cheaguri de sânge.	○	○	○
K4. Doar persoanele în vârstă pot dezvolta tromboză venoasă.	○	○	○
K5. Obezitatea crește riscul de tromboză.	○	○	○
K6. Fumatul nu are nicio influență asupra riscului de cheaguri de sânge.	○	○	○
K7. Călătoriile lungi (peste 4 ore) cu avionul sau mașina pot crește riscul de cheaguri de sânge.	○	○	○
K8. Contraceptivele hormonale pot crește riscul de tromboză la femei.	○	○	○
K9. Cancerul nu are niciun efect asupra riscului de cheaguri de sânge.	○	○	○
K10. Sarcina și primele săptămâni după naștere cresc riscul de tromboză.	○	○	○
K11. Durerea și umflarea bruscă a unei gambe pot fi semne de tromboză venoasă profundă.	○	○	○
K12. Lipsa bruscă de aer și durerea în piept pot fi semne de trombembolism pulmonar.	○	○	○
K13. Activitatea fizică regulată crește riscul de tromboză.	○	○	○
K14. Un consum ridicat de carne roșie și mezeluri se poate asocia cu un risc mai mare de tromboză.	○	○	○
K15. Trombembolismul pulmonar nu pune niciodată viața în pericol.	○	○	○

Scor cunoștințe (investigator): 1 punct pentru fiecare răspuns corect; «Nu știu» = 0 puncte (total 0–15).

Secțiunea F. Activitate fizică — Chestionarul internațional de activitate fizică (IPAQ, forma scurtă)

Întrebările se referă la timpul petrecut în activitate fizică în ultimele 7 zile. Luați în calcul doar activitățile efectuate cel puțin 10 minute fără întrerupere.

F1. În ultimele 7 zile, în câte zile ați efectuat activități fizice INTENSE (efort mare, respirație mult accelerată — ex. ridicat greutăți mari, săpat, alergare, aerobic, ciclism rapid)?

zile pe săptămână: ______ sau ☐ niciuna → treceți la întrebarea F3

F2. În acele zile, cât timp ați petrecut în medie pe zi cu activități intense?

ore: ______ minute: ______

F3. În ultimele 7 zile, în câte zile ați efectuat activități fizice MODERATE (efort moderat, respirație ceva mai accelerată — ex. cărat greutăți ușoare, ciclism în ritm obișnuit)? NU includeți mersul pe jos.

zile pe săptămână: ______ sau ☐ niciuna → treceți la întrebarea F5

F4. În acele zile, cât timp ați petrecut în medie pe zi cu activități moderate?

ore: ______ minute: ______

F5. În ultimele 7 zile, în câte zile ați MERS PE JOS cel puțin 10 minute fără oprire (inclusiv la serviciu, acasă, ca mijloc de deplasare, sport sau recreere)?

zile pe săptămână: ______ sau ☐ niciuna → treceți la întrebarea F7

F6. În acele zile, cât timp ați petrecut în medie pe zi mergând pe jos?

ore: ______ minute: ______

F7. Într-o zi lucrătoare obișnuită, cât timp ați petrecut STÂND JOS / așezat (la birou, masă, TV, transport)?

ore: ______ minute: ______

Cotare (investigator): MET-minute/săpt = mers 3,3 × min × zile + moderat 4,0 × min × zile + intens 8,0 × min × zile. Prelucrare: episoadele < 10 min nu se iau în calcul; fiecare tip de activitate se trunchiază la 180 min/zi; se exclud cazurile cu un total > 960 min/zi. Categorii: Ridicat (activitate intensă în ≥ 3 zile, totalizând ≥ 1.500 MET-min/săpt; SAU ≥ 7 zile cu orice combinație de mers, activitate moderată sau intensă, totalizând ≥ 3.000 MET-min/săpt) / Moderat (criteriile pentru Ridicat nu sunt îndeplinite și: ≥ 3 zile de activitate intensă ≥ 20 min/zi; SAU ≥ 5 zile de activitate moderată și/sau mers ≥ 30 min/zi; SAU ≥ 5 zile cu orice combinație, totalizând ≥ 600 MET-min/săpt) / Scăzut (niciun criteriu îndeplinit).

Secțiunea G. Obiceiuri alimentare — Scor de aderență la dieta mediteraneană (MEDAS, 14 itemi)

Vă rugăm să vă gândiți la obiceiurile alimentare din ultima perioadă. Bifați varianta care descrie cel mai bine obiceiul dvs. Varianta marcată «(1p)» indică răspunsul care primește 1 punct.

G1. Folosiți uleiul de măsline ca principală grăsime pentru gătit?
☐ Da (1p) ☐ Nu
G2. Ce cantitate de ulei de măsline consumați în total pe zi (gătit, salate, mese în oraș)? (1 lingură ≈ 10 ml)
☐ ≥ 4 linguri (1p) ☐ < 4 linguri
G3. Câte porții de legume consumați pe zi? (1 porție = 200 g; garnitura = ½ porție)
☐ ≥ 2 porții, din care ≥ 1 crude/salată (1p) ☐ < 2 porții
G4. Câte fructe (inclusiv suc natural de fructe) consumați pe zi?
☐ ≥ 3 (1p) ☐ < 3
G5. Câte porții de carne roșie, hamburger sau mezeluri consumați pe zi? (1 porție = 100–150 g)
☐ < 1 (1p) ☐ ≥ 1
G6. Câte porții de unt, margarină sau smântână consumați pe zi? (1 porție = 12 g)
☐ < 1 (1p) ☐ ≥ 1
G7. Câte băuturi dulci sau carbogazoase consumați pe zi?
☐ < 1 (1p) ☐ ≥ 1
G8. Cât vin consumați pe săptămână? (Item MEDAS original; consumul de alcool este înregistrat și separat — Secțiunea I — și se interpretează cu prudență.)
☐ ≥ 7 pahare (1p) ☐ < 7 pahare
G9. Câte porții de leguminoase (fasole, mazăre, linte, năut) consumați pe săptămână? (1 porție = 150 g)
☐ ≥ 3 (1p) ☐ < 3
G10. Câte porții de pește sau fructe de mare consumați pe săptămână? (100–150 g pește / 200 g fructe de mare)
☐ ≥ 3 (1p) ☐ < 3
G11. De câte ori pe săptămână consumați dulciuri sau produse de patiserie comerciale (prăjituri, biscuiți, checuri)?
☐ < 3 (1p) ☐ ≥ 3
G12. Câte porții de nuci sau alune (inclusiv arahide) consumați pe săptămână? (1 porție = 30 g)
☐ ≥ 3 (1p) ☐ < 3
G13. Preferați carnea de pui, curcan sau iepure în locul cărnii de vită, porc, hamburger sau cârnați?
☐ Da (1p) ☐ Nu
G14. De câte ori pe săptămână consumați legume, paste, orez sau alte preparate gătite cu sos «sofrito» (roșii cu ceapă/usturoi/praz călite în ulei de măsline)?
☐ ≥ 2 (1p) ☐ < 2
Scor MEDAS total (investigator, 0–14): ______ | Interpretare: ≤ 5 aderență scăzută; 6–9 moderată; ≥ 10 aderență ridicată.

Secțiunea H. Fumat — status și Testul Fagerström de dependență de nicotină (FTND)

H1. Care este statutul dvs. de fumător?
☐ Nefumător (niciodată) ☐ Fost fumător ☐ Fumător actual
Dacă fost fumător, de câți ani ați renunțat? ______ ani
Număr de țigări pe zi (actual sau în perioada de fumat): ______ țigări/zi
Număr total de ani de fumat: ______ ani → Pachete-an (investigator: nr. țigări/zi ÷ 20 × ani): ______
Întrebările FTND de mai jos se completează DOAR de fumătorii actuali. Bifați răspunsul; punctajul este notat între paranteze.
H2.1. Cât de curând după trezire fumați prima țigară?
☐ în ≤ 5 min (3) ☐ 6–30 min (2) ☐ 31–60 min (1) ☐ după 60 min (0)
H2.2. Vă este greu să vă abțineți de la fumat în locuri unde este interzis (biserică, cinema, tren, spital)?
☐ Da (1) ☐ Nu (0)
H2.3. La ce țigară v-ar fi cel mai greu să renunțați?
☐ Prima de dimineață (1) ☐ Oricare alta (0)
H2.4. Câte țigări fumați pe zi?
☐ ≤ 10 (0) ☐ 11–20 (1) ☐ 21–30 (2) ☐ ≥ 31 (3)
H2.5. Fumați mai des în primele ore după trezire decât în restul zilei?
☐ Da (1) ☐ Nu (0)
H2.6. Fumați chiar dacă sunteți atât de bolnav încât stați în pat aproape toată ziua?
☐ Da (1) ☐ Nu (0)
Scor FTND total (investigator, 0–10): ______ | Interpretare: 0–2 foarte scăzut; 3–4 scăzut; 5 moderat; 6–7 ridicat; 8–10 foarte ridicat.

Secțiunea I. Consum de alcool

I1. Consumați băuturi alcoolice?
☐ Nu, niciodată ☐ Da

I2. Cât de frecvent consumați alcool?
☐ Ocazional (< 1/săpt) ☐ 1–3 ori/săpt ☐ 4–6 ori/săpt ☐ Zilnic

I3. Număr aproximativ de unități de alcool pe săptămână: ______ unități

1 unitate ≈ 12 g alcool pur ≈ 330 ml bere (5%) ≈ 125 ml vin (12%) ≈ 40 ml băutură spirtoasă (40%).

Vă mulțumim pentru timpul acordat!

Fișă de scor — se completează de investigator (nu se înmânează participantului)

1. Scor cunoștințe (0–15). Cheie de corectare: ADEVĂRAT pentru K1–K3, K5, K7, K8, K10–K12, K14; FALS pentru K4, K6, K9, K13, K15; «Nu știu» = 0 puncte. Interpretare provizorie: < 8 scăzut; 8–11 moderat; ≥ 12 ridicat; cunoștințe adecvate = ≥ 11 puncte (≥ 70%). Scor: ______

2. Scoruri atitudini. Subscala «amenințare percepută» = suma AT1–AT4 (4–20): ______; subscala «beneficii percepute și autoeficacitate» = suma AT5–AT8 (4–20): ______. Interpretare provizorie pe subscală: ≥ 16 favorabil; 12–15 neutru-pozitiv; < 12 nefavorabil. Nu se calculează un scor total de atitudine. AT9–AT11 se raportează individual (1–5). SC1 nu se punctează.

3. Indice practici (0–12). PR1–PR6 cotate 2/1/0 în ordinea variantelor listate și însumate; dacă lipsește un singur item, totalul se proratează (media celor 5 itemi × 6); cu ≥ 2 itemi lipsă, indicele nu se calculează. Se exprimă și procentual: ≥ 75% bun; 50–74% moderat; < 50% limitat. PR7–PR8 (situaționale) nu intră în indice și se raportează descriptiv. Scor: ______ (______ %)

4. Module validate. Categorie IPAQ-SF: ☐ scăzut ☐ moderat ☐ ridicat | MEDAS (0–14): ______ (☐ scăzută ☐ moderată ☐ ridicată) | FTND (0–10, doar fumători actuali): ______ | Pachete-an: ______

5. Indice exploratoriu al încărcăturii cu factori de risc TEV (neponderat). Se numără separat elementele prezente din fiecare domeniu; «Nu știu» = absent (0).

Element (item-sursă)	Domeniu	Prezent? (bifați)
Antecedente personale de TVP/TEP (B1)	Clinic	☐
Trombofilie cunoscută (B3)	Clinic	☐
Cancer activ sau tratat în ultimii 5 ani (B4)	Clinic	☐
Chirurgie majoră / traumatism în ultimele 3 luni (B5)	Clinic	☐
Imobilizare/spitalizare ≥ 3 zile în ultimele 3 luni (B6)	Clinic	☐
Vârsta ≥ 60 ani (A1)	Clinic	☐
Antecedente familiale de TEV, rude de gradul I (B2)	Clinic	☐
Terapie hormonală sau sarcină/lăuzie (B8)	Clinic	☐
Călătorie lungă > 4 ore în ultima lună (B7)	Clinic (situațional)	☐
Varice / insuficiență venoasă cronică (B9)	Clinic	☐
Obezitate, IMC ≥ 30 kg/m² (A6)	Stil de viață	☐
Fumat actual (H1)	Stil de viață	☐
Activitate fizică scăzută (IPAQ categoria scăzut)	Stil de viață	☐
Aderență scăzută la dieta mediteraneană (MEDAS ≤ 5)	Stil de viață	☐

Cele două domenii se raportează separat și nu se însumează; nu se definesc categorii de risc. Elementele nu sunt ponderate, iar un număr egal de factori nu înseamnă o importanță clinică egală. Indicele este descriptiv, nu estimează probabilitatea de TEV, nu este un instrument de diagnostic sau prognostic și nu înlocuiește evaluarea medicală.

Număr de factori — domeniul clinic/situațional (0–10): ______ domeniul stil de viață (0–4): ______
