# Supplementary material for: Development and Psychometric Validation of a Knowledge, Attitudes, and Practices Questionnaire on Pulmonary Embolism and Its Modifiable Risk Factors (PE-KAP-RO): Protocol for a Cross-Sectional Study in Adults from Western Romania
Source: Healthcare (Basel). 2026 Oct 23;14(21):3599. doi: 10.3390/healthcare14213599 (PMC13762760; doi:10.3390/healthcare14213599)
Supplement: Supplementary file 1 [file healthcare-14-03599-s001.zip › healthcare-4553484_Supplementary_File_S2.pdf]

PE-KAP-RO QUESTIONNAIRE on knowledge, attitudes, and practices regarding pulmonary embolism and its risk factors — English translation

Supplementary File S2. English translation of the participant form provided as Supplementary File S1 (obtained by forward–backward translation; the instrument is administered in Romanian)

Thank you for taking part. Completion is voluntary and anonymous and takes approximately 15 minutes. There are no "right" or "wrong" answers to the questions about attitudes and habits—please answer honestly. Data are used exclusively for research, processed in aggregate, in accordance with Regulation (EU) 2016/679 (GDPR).

Informed consent

I have read and understood the information above on the purpose of the study and how data are processed. I understand that participation is voluntary and anonymous, that my answers contain no directly identifying data, and that I may stop completing the questionnaire at any time without any consequence. Data controller: "Victor Babeș" University of Medicine and Pharmacy Timișoara; processing complies with Regulation (EU) 2016/679 (GDPR).

☐ YES, I agree to take part in this study and for the data I provide to be used for scientific research, including publication of the results in aggregated and anonymized form.

☐ YES, I agree to be re-contacted for a second completion of the questionnaire (test–retest, after approximately 14 days) — optional. Contact (phone/e-mail, optional): ______________________ (contact details are stored separately from the answers and deleted after the second completion)

Questionnaires without the first consent box ticked will not be processed.

Section A. Sociodemographic and Anthropometric Data

Participant code (investigator): ________ Date of completion: ____ / ____ / ________

A1. Age (completed years): ________

A2. Sex:
☐ Female ☐ Male

A3. Area of residence:
☐ Urban ☐ Rural

A4. Educational level:
☐ No formal / primary education ☐ Lower secondary ☐ Upper secondary / post-secondary ☐ University / postgraduate

A5. Occupational status / type of activity:
☐ Predominantly sedentary (desk) work ☐ Predominantly physical work ☐ Unemployed ☐ Retired ☐ Student ☐ Homemaker

A6. Height: ______ cm Current weight: ______ kg BMI (investigator, kg/m^2): ______

A7. Waist circumference (if known): ______ cm

A8. Do you work or study in the healthcare field?
☐ No ☐ Yes — physician ☐ Yes — nurse, midwife, or other health professional ☐ Yes — pharmacist ☐ Yes — student in a health-related field

Section B. Personal History and Risk Factors

Please tick what applies to you. These data are self-reported.

B1. Have you ever been diagnosed with venous thrombosis (DVT) or pulmonary embolism (PE)?
☐ Yes ☐ No ☐ Don't know

B2. Has a first-degree relative (parent, sibling, child) had venous thrombosis / PE?
☐ Yes ☐ No ☐ Don't know

B3. Do you have a known thrombophilia (a diagnosed clotting disorder)?
☐ Yes ☐ No ☐ Don't know

B4. Cancer (active, or treated within the last 5 years)?
☐ Yes ☐ No

B5. Major surgery, or trauma / fracture, in the last 3 months?
☐ Yes ☐ No

B6. Prolonged immobilization or hospitalization (≥ 3 days in bed) in the last 3 months?
☐ Yes ☐ No

B7. Long-distance travel (> 4 hours, by plane or car) in the last month?
☐ Yes ☐ No

B8. Women only — hormonal therapy / pregnancy:
☐ Hormonal contraceptives ☐ Hormone replacement therapy ☐ Pregnancy / postpartum (last 6 weeks) ☐ None / not applicable

B9. Known chronic conditions (tick all that apply):
☐ Hypertension ☐ Diabetes mellitus ☐ Dyslipidemia ☐ Heart disease ☐ Chronic respiratory disease ☐ Chronic kidney disease ☐ Varicose veins / chronic venous insufficiency ☐ None

B10. Are you currently taking anticoagulant or antiplatelet treatment?
☐ Yes ☐ No ☐ Don't know

Section C. Awareness and Attitudes

SC1. Have you ever heard of deep vein thrombosis (DVT) or pulmonary embolism (PE)?
☐ Yes ☐ No

Please indicate how much you agree with each statement (1 = strongly disagree ... 5 = strongly agree).

Statement	1 Strongly Disagree	2	3 Neutral	4	5 Strongly Agree
AT1. I consider thrombosis / pulmonary embolism to be a serious condition.	○	○	○	○	○
AT2. If I developed a blood clot, it would seriously affect my health and daily life.	○	○	○	○	○
AT3. I believe that I personally could be at risk of thrombosis.	○	○	○	○	○
AT4. Given my health and lifestyle, it is possible that I will develop a blood clot at some point in my life.	○	○	○	○	○
AT5. Preventing thrombosis is important for my health.	○	○	○	○	○
AT6. Taking preventive measures in daily life would lower my chance of developing a blood clot.	○	○	○	○	○
AT7. I feel able to take concrete preventive measures in my daily life.	○	○	○	○	○
AT8. I am confident that I could change my daily habits if a doctor told me I was at risk of thrombosis.	○	○	○	○	○
AT9. I am willing to change my lifestyle to reduce my risk of thrombosis.	○	○	○	○	○
AT10. I would like to receive more information about preventing thrombosis.	○	○	○	○	○
AT11. If I noticed symptoms that could indicate a blood clot, I would seek medical care quickly.	○	○	○	○	○

Section D. Preventive Practices and Behaviors

PR1. When you sit for a long time (at work, at home, in front of a screen), how often do you get up and move around at least once every hour?
☐ Usually ☐ Sometimes ☐ Rarely or never

PR2. When you have to stay seated for a long time, do you move or flex your feet and legs?
☐ Usually ☐ Sometimes ☐ Rarely or never

PR3. Do you monitor your body weight?
☐ Yes, regularly ☐ Occasionally ☐ No

PR4. How often do you see a doctor for a check-up?
☐ At least once a year ☐ Less than once a year ☐ Never

PR5. Have you ever looked for information about preventing blood clots (doctor, leaflets, internet)?
☐ Several times ☐ Once or twice ☐ Never

PR6. Have you ever talked with a doctor, nurse, or pharmacist about your own risk of blood clots (for example, when starting a hormonal treatment, before surgery, or at a check-up)?
☐ Yes, more than once ☐ Yes, once ☐ No

The next two questions apply only to some situations; tick "not applicable" if the situation does not apply to you.

PR7. On long journeys (> 4 hours), do you usually get up / stop and move around at regular intervals?
☐ Always ☐ Sometimes ☐ Never ☐ Not applicable (I do not make long journeys)

PR8. When prevention of thrombosis was recommended during a previous hospitalization or operation (injections and/or compression stockings), did you follow it?
☐ Yes, completely ☐ Only partly ☐ No, I did not follow it ☐ Not applicable (never hospitalized / never recommended)

Section E. Knowledge About Thrombosis and Pulmonary Embolism

For each statement, mark whether you consider it true or false, or tick "Don't know".

Statement	True	False	Don't Know
K1. A pulmonary embolism is a blood clot that blocks the arteries of the lungs.	○	○	○
K2. Deep vein thrombosis most often develops in the veins of the legs.	○	○	○
K3. Prolonged immobilization (bed rest, sitting still for long periods) increases the risk of blood clots.	○	○	○
K4. Only elderly people can develop venous thrombosis.	○	○	○
K5. Obesity increases the risk of thrombosis.	○	○	○
K6. Smoking has no influence on the risk of blood clots.	○	○	○
K7. Long journeys (over 4 hours) by plane or car can increase the risk of blood clots.	○	○	○
K8. Hormonal contraceptives can increase the risk of thrombosis in women.	○	○	○
K9. Cancer has no effect on the risk of blood clots.	○	○	○
K10. Pregnancy and the first weeks after childbirth increase the risk of thrombosis.	○	○	○
K11. Pain and sudden swelling of one calf can be signs of deep vein thrombosis.	○	○	○
K12. Sudden shortness of breath and chest pain can be signs of pulmonary embolism.	○	○	○
K13. Regular physical activity increases the risk of thrombosis.	○	○	○
K14. A diet high in red and processed meat may be associated with a higher risk of thrombosis.	○	○	○
K15. A pulmonary embolism is never life-threatening.	○	○	○

Knowledge score (investigator): 1 point per correct answer; "don't know" = 0 points (total 0–15).

Section F. Physical Activity — International Physical Activity Questionnaire, Short Form (IPAQ-SF)

The questions refer to time spent in physical activity during the last 7 days. Count only activities performed for at least 10 minutes without interruption.

F1. During the last 7 days, on how many days did you do VIGOROUS physical activities (hard effort, much faster breathing — e.g., heavy lifting, digging, running, aerobics, fast cycling)?

days per week: ______ or ☐ none → go to question F3

F2. On those days, how much time did you usually spend per day on vigorous activities?

hours: ______ minutes: ______

F3. During the last 7 days, on how many days did you do MODERATE physical activities (moderate effort, somewhat faster breathing — e.g., carrying light loads, cycling at a regular pace)? Do NOT include walking.

days per week: ______ or ☐ none → go to question F5

F4. On those days, how much time did you usually spend per day on moderate activities?

hours: ______ minutes: ______

F5. During the last 7 days, on how many days did you WALK for at least 10 minutes at a time (at work, at home, for transport, sport, or leisure)?

days per week: ______ or ☐ none → go to question F7

F6. On those days, how much time did you usually spend walking per day?

hours: ______ minutes: ______

F7. On a usual weekday, how much time did you spend SITTING (desk, table, TV, transport)?

hours: ______ minutes: ______

Scoring (investigator): MET-min/week = walking 3.3 × min × days + moderate 4.0 × min × days + vigorous 8.0 × min × days. Data processing: bouts $<$ 10 min excluded; each activity type truncated at 180 min/day; cases with a total $>$ 960 min/day excluded. Categories: High (vigorous activity on $\geq$ 3 days accumulating $\geq$ 1,500 MET-min/week; OR $\geq$ 7 days of any combination of walking, moderate, or vigorous activity accumulating $\geq$ 3,000 MET-min/week) / Moderate (not High, and $\geq$ 3 days of vigorous activity $\geq$ 20 min/day; OR $\geq$ 5 days of moderate activity and/or walking $\geq$ 30 min/day; OR $\geq$ 5 days of any combination accumulating $\geq$ 600 MET-min/week) / Low (neither criterion met).

Section G. Dietary Habits — Mediterranean Diet Adherence Screener (MEDAS, 14 Items)

Please think of your usual eating habits over the recent period. Tick the option that best describes your habit. The option marked "(1p)" receives 1 point.

G1. Do you use olive oil as your main fat for cooking?

☐ Yes (1p) ☐ No

G2. How much olive oil do you consume in total per day (cooking, salads, meals away from home)? (1 tablespoon ≈ 10 mL)

☐ ≥ 4 tablespoons (1p) ☐ < 4 tablespoons

G3. How many servings of vegetables do you consume per day? (1 serving = 200 g; a side dish = ½ serving)

☐ ≥ 2 servings, of which ≥ 1 raw or as salad (1p) ☐ < 2 servings

G4. How many pieces of fruit (including natural fruit juice) do you consume per day?

☐ ≥ 3 (1p) ☐ < 3

G5. How many servings of red meat, hamburger, or processed meat do you consume per day? (1 serving = 100–150 g)

☐ < 1 (1p) ☐ ≥ 1

G6. How many servings of butter, margarine, or cream do you consume per day? (1 serving = 12 g)

☐ < 1 (1p) ☐ ≥ 1

G7. How many sweetened or carbonated beverages do you drink per day?

☐ < 1 (1p) ☐ ≥ 1

G8. How much wine do you drink per week? (Original MEDAS item; alcohol intake is also recorded separately in Section I and interpreted with caution.)

☐ ≥ 7 glasses (1p) ☐ < 7 glasses

G9. How many servings of legumes (beans, peas, lentils, chickpeas) do you consume per week? (1 serving = 150 g)

☐ ≥ 3 (1p) ☐ < 3

G10. How many servings of fish or seafood do you consume per week? (100–150 g fish / 200 g seafood)

☐ ≥ 3 (1p) ☐ < 3

G11. How many times per week do you consume commercial sweets or pastries (cakes, biscuits, sponge cakes)?

☐ < 3 (1p) ☐ ≥ 3

G12. How many servings of nuts (including peanuts) do you consume per week? (1 serving = 30 g)

☐ ≥ 3 (1p) ☐ < 3

G13. Do you prefer chicken, turkey, or rabbit instead of beef, pork, hamburger, or sausages?

☐ Yes (1p) ☐ No

G14. How many times per week do you consume vegetables, pasta, rice, or other dishes prepared with a "sofrito" sauce (tomatoes with onion/garlic/leek simmered in olive oil)?

☐ ≥ 2 (1p) ☐ < 2

Total MEDAS score (investigator, 0–14): ______ | Interpretation: ≤ 5 low adherence; 6–9 moderate; ≥ 10 high adherence.

Section H. Smoking — Status and Fagerström Test for Nicotine Dependence (FTND)

H1. What is your smoking status?

☐ Never smoker ☐ Former smoker ☐ Current smoker

If former smoker, how many years since you quit? ______ years

Number of cigarettes per day (currently, or during the smoking period): ______ cigarettes/day

Total years of smoking: ______ years → Pack-years (investigator: cigarettes/day ÷ 20 × years): ______

The FTND questions below are completed ONLY by current smokers. Tick one answer; the score is shown in parentheses.

H2.1. How soon after waking do you smoke your first cigarette?

☐ Within 5 min (3) ☐ 6–30 min (2) ☐ 31–60 min (1) ☐ After 60 min (0)

H2.2. Do you find it difficult to refrain from smoking in places where it is forbidden (church, cinema, train, hospital)?

☐ Yes (1) ☐ No (0)

H2.3. Which cigarette would you hate most to give up?

☐ The first one in the morning (1) ☐ Any other (0)

H2.4. How many cigarettes do you smoke per day?

☐ ≤ 10 (0) ☐ 11–20 (1) ☐ 21–30 (2) ☐ ≥ 31 (3)

H2.5. Do you smoke more frequently during the first hours after waking than during the rest of the day?

☐ Yes (1) ☐ No (0)

H2.6. Do you smoke even when you are so ill that you are in bed most of the day?

☐ Yes (1) ☐ No (0)

Total FTND score (investigator, 0–10): ______ | Interpretation: 0–2 very low; 3–4 low; 5 moderate; 6–7 high; 8–10 very high dependence.

Section I. Alcohol Intake

I1. Do you drink alcoholic beverages?

☐ No, never ☐ Yes

I2. How often do you drink alcohol?

☐ Occasionally (< 1/week) ☐ 1–3 times/week ☐ 4–6 times/week ☐ Daily

I3. Approximate number of units of alcohol per week: ______ units

1 unit ≈ 12 g pure alcohol ≈ 330 mL beer (5%) ≈ 125 mL wine (12%) ≈ 40 mL spirits (40%).

Thank you for your time!

Score sheet — completed by the investigator (not handed to the participant)

1. Knowledge score (0–15). Answer key: TRUE for K1–K3, K5, K7, K8, K10–K12, K14; FALSE for K4, K6, K9, K13, K15; "Don't know" = 0 points. Provisional interpretation: < 8 low; 8–11 moderate; ≥ 12 high; adequate knowledge = ≥ 11 points (≥ 70%). Score: ______

2. Attitude scores. Perceived threat subscale = sum of AT1–AT4 (4–20): ______; perceived benefits and self-efficacy subscale = sum of AT5–AT8 (4–20): ______. Provisional interpretation per subscale: ≥ 16 favorable; 12–15 neutral-to-positive; < 12 unfavorable. No total attitude score is computed. AT9–AT11 are reported individually (1–5). SC1 is not scored.

3. Practice index (0–12). PR1–PR6 scored 2/1/0 in the order the options are listed and summed; if a single item is missing, the total is prorated (mean of the 5 answered items × 6); with ≥ 2 items missing, the index is not computed. Also expressed as a percentage: ≥ 75% good; 50–74% moderate; < 50% limited. PR7–PR8 (situation-specific) are not part of the index and are reported descriptively. Score: ______ (______ %)

4. Validated modules. IPAQ-SF category: ☐ low ☐ moderate ☐ high | MEDAS (0–14): ______ (☐ low ☐ moderate ☐ high) | FTND (0–10, current smokers only): ______ | Pack-years: ______

5. Exploratory VTE risk-factor burden index (unweighted). Count the elements present separately for each domain; "Don't know" = absent (0).

Element (source item)	Domain	Present? (tick)
Personal history of DVT/PE (B1)	Clinical	☐
Known thrombophilia (B3)	Clinical	☐
Active cancer, or treated within the last 5 years (B4)	Clinical	☐
Major surgery / trauma in the last 3 months (B5)	Clinical	☐
Immobilization/hospitalization ≥ 3 days in the last 3 months (B6)	Clinical	☐
Age ≥ 60 years (A1)	Clinical	☐
First-degree family history of VTE (B2)	Clinical	☐
Hormonal therapy or pregnancy/postpartum (B8)	Clinical	☐
Long-distance travel > 4 h in the last month (B7)	Clinical (situational)	☐
Varicose veins / chronic venous insufficiency (B9)	Clinical	☐
Obesity, BMI ≥ 30 kg/m² (A6)	Lifestyle	☐
Current smoking (H1)	Lifestyle	☐
Low physical activity (IPAQ-SF category low)	Lifestyle	☐
Low Mediterranean diet adherence (MEDAS ≤ 5)	Lifestyle	☐

The two domains are reported separately and are not summed; no risk categories are defined. Elements are unweighted, and an equal number of factors does not imply equal clinical importance. The index is descriptive, does not estimate the probability of VTE, is not a diagnostic or prognostic instrument, and does not replace medical evaluation.

Number of factors — clinical/situational domain (0–10): ______ lifestyle domain (0–4): ______
